# Manipulation of charge carrier flow in Bi_4_NbO_8_Cl nanoplate photocatalyst with metal loading†

**DOI:** 10.1039/d1sc06054f

**Published:** 2022-01-24

**Authors:** Kanta Ogawa, Ryota Sakamoto, Chengchao Zhong, Hajime Suzuki, Kosaku Kato, Osamu Tomita, Kouichi Nakashima, Akira Yamakata, Takashi Tachikawa, Akinori Saeki, Hiroshi Kageyama, Ryu Abe

**Affiliations:** Department of Energy and Hydrocarbon Chemistry, Graduate School of Engineering, Kyoto University Nishikyo-ku Kyoto 615-8510 Japan; Graduate School of Engineering, Toyota Technological Institute 2-12-1 Hisakata, Tempaku Nagoya 468-8511 Japan; Department of Materials Science and Engineering, College of Engineering, Ibaraki University 4-12-1, Nakanarusawa Hitachi Ibaraki 316-8511 Japan; Molecular Photoscience Research Center, Kobe University 1-1 Rokkodai-cho Kobe 657-8501 Japan; Department of Applied Chemistry, Graduate School of Engineering, Osaka University 2-1 Yamadaoka Suita Osaka 565-0871 Japan

## Abstract

Separation of photoexcited charge carriers in semiconductors is important for efficient solar energy conversion and yet the control strategies and underlying mechanisms are not fully established. Although layered compounds have been widely studied as photocatalysts, spatial separation between oxidation and reduction reaction sites is a challenging issue due to the parallel flow of photoexcited carriers along the layers. Here we demonstrate orthogonal carrier flow in layered Bi_4_NbO_8_Cl by depositing a Rh cocatalyst at the edges of nanoplates, resulting in spatial charge separation and significant enhancement of the photocatalytic activity. Combined experimental and theoretical studies revealed that lighter photogenerated electrons, due to a greater in-plane dispersion of the conduction band (*vs.* valence band), can travel along the plane and are readily trapped by the cocatalyst, whereas the remaining holes hop perpendicular to the plane because of the anisotropic crystal geometry. Our results propose manipulating carrier flow *via* cocatalyst deposition to achieve desirable carrier dynamics for photocatalytic reactions in layered compounds.

## Abstract 2

An efficient spatial separation of photoexcited carriers is demonstrated in a layered-oxyhalide nanoplate by controlling the direction of carrier flows upon Rh cocatalyst loading, which leads to drastic photocatalytic H_2_ evolution activity.

## Graphical abstract

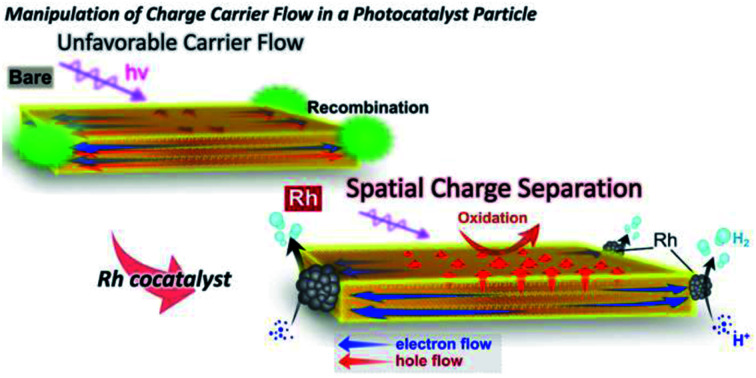

## Introduction

Semiconductor photocatalysis is a promising solar energy conversion method. Therein, photogenerated charge carriers migrate from the bulk to the surface to induce redox reactions – water splitting being the primary target.^1–3^ For practical solar-to-hydrogen conversion systems *via* photocatalytic water splitting, both the effective absorption of photons in the visible light region and the efficient charge separation of photoexcited charge carriers (electrons and holes) are crucial. Thus, semiconductor materials with narrow band gaps such as non-oxides and mixed-anion materials have increasingly been investigated as photocatalysts.^4–9^ Although an extraordinarily high quantum efficiency of nearly 100% has recently been demonstrated under UV light on an Al-doped SrTiO_3_ particulate photocatalyst,^3^ such highly efficient charge separation has not yet been achieved on visible-light-responsive photocatalysts. Recent efforts, including the Al-doped SrTiO_3_ and some visible-responsive materials (*e.g.*, BiVO_4_, BaTaO_2_N), have focused on facet engineering to expose crystal faces suitable for redox reactions,^3,10–14^ where the energy level of each facet determines the charge separation. However, it is not always possible to expose a desired facet of the targeted semiconductors. Moreover, this strategy relies on the intrinsic characteristics of photocatalysts' facets and does not answer the fundamental question of how charge separation is achieved in the bulk of photocatalysts; the carrier flow inside the photocatalyst particle is still not fully understood.

Layered semiconductors such as UV-responsive K_4_Nb_6_O_17_ have shown promise in photocatalysis^4,15,16^ with a couple of advantages, including their controllability of the chemical composition through ion exchange^17–19^ and large surface-to-volume ratios.^20^ However, from the viewpoint of efficient charge separation inside the photocatalysts, two-dimensional crystal structures may be unfavorable because both photoexcited electrons and holes travel mainly along the in-plane direction, as observed theoretically^21–24^ and experimentally.^25–27^ Such parallel carrier flows based on the crystal and band structure make it difficult to separate the oxidation and reduction reaction sites in the photocatalyst particles, often resulting in charge recombination. Although the reduction and oxidation sites on several layered photocatalysts such as BaLa_4_Ti_4_O_15_ were separated,^13,28^ the underlying carrier flows in the bulk of the photocatalysts remain elusive.

**Fig. 1:**
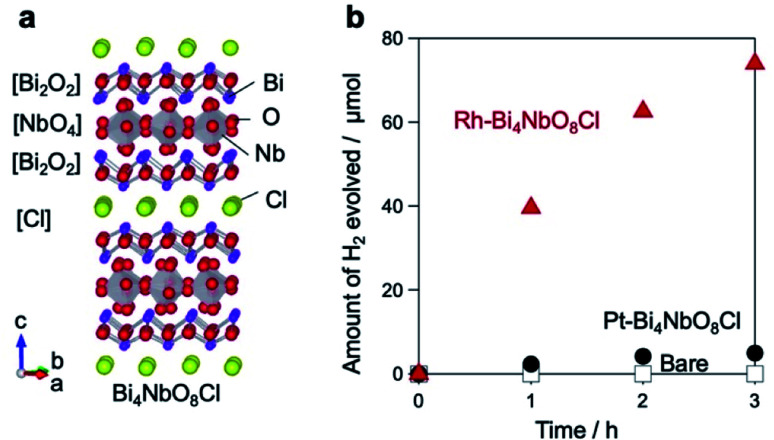
(a) Crystal structure of the layered perovskite oxyhalide Bi_4_NbO_8_Cl. (b) Time courses of H_2_ evolution over Bare-Bi_4_NbO_8_Cl (squares), Rh-Bi_4_NbO_8_Cl (triangles) and Pt-Bi_4_NbO_8_Cl (circles) in a methanol aqueous solution (20 vol%) under photoirradiation (*λ* > 300 nm).

We have recently reported that Bi_4_NbO_8_Cl, with a layered Sillén–Aurivillius structure comprising [Bi_2_O_2_], [NbO_4_], and [Cl] modules (Fig. 1a), exhibits the highest activity among layered compounds, as an O_2_ evolution photocatalyst in visible-light Z-scheme water-splitting.^29^ When prepared by a flux method, Bi_4_NbO_8_Cl yields as nanoplates with good crystallinity.^30^ However, despite its band positions being suitable for both H_2_ and O_2_ production from water, the H_2_ evolution activity of Bi_4_NbO_8_Cl was negligible, and surprisingly, the situation did not change when Pt, an established H_2_ evolution cocatalyst,^4^ was introduced.^31,32^ This result suggests that charge carriers are not effectively separated in Bi_4_NbO_8_Cl.

Here, we demonstrate an efficient spatial charge separation in Bi_4_NbO_8_Cl nanoplates by controlling the direction of carrier flows with Rh loading. In the absence of a cocatalyst, both photoexcited electrons and holes travel along the in-plane direction based on the band dispersions, resulting in recombination at the edge of the nanoplate. However, site-selective Rh deposition at the edge allows the electrons to migrate in the in-plane direction and to be captured by the cocatalyst, while the holes hop along the out-of-plane direction. The resultant spatial charge separation significantly improves the H_2_ evolution activity. Based on experimental and theoretical investigations, we discuss the carrier flow dynamics and the associated photocatalytic properties of Rh-loaded Bi_4_NbO_6_Cl nanoplates in comparison to unloaded- and Pt-loaded ones.

## Results and discussion

### Improvement of Bi_4_NbO_8_Cl photocatalytic H_2_ evolution activity

As stated above, the H_2_ evolution activity of bare Bi_4_NbO_8_Cl (hereafter denoted as Bare-Bi_4_NbO_8_Cl) is low even though it has a conduction band (CB) potential sufficient for H^+^ reduction,^31^ and little improvement is seen when the Pt cocatalyst (5 wt%) is loaded by a photodeposition (PD) method (Pt-Bi_4_NbO_8_Cl; Fig. 1b). In contrast, Rh loading (5 wt%) *via* PD significantly enhances photocatalytic H_2_ production, as displayed in the time course of H_2_ evolution (Rh-Bi_4_NbO_8_Cl; Fig. 1b). We additionally investigated H_2_ production activities for samples with various metal cocatalysts (Pt, Ru, Ir, Pd, Au, Rh) deposited by PD and impregnation methods, the most frequently used methods for particulate photocatalysts. The results highlight the superiority of Rh-Bi_4_NbO_8_Cl (Fig. S1†). H_2_ evolution is observed at a remarkably high rate under visible-light irradiation (*λ* > 400 nm) on Rh-Bi_4_NbO_8_Cl (Fig. S2†). This result confirms that visible-light water splitting is possible and that photocatalytic H_2_ evolution is based on the bandgap excitation of Bi_4_NbO_8_Cl (Fig. S3†). Although the activity decreased during long-term irradiation due to physical detachment of the cocatalyst (Fig. S4†), the stability will be improved further by elaborated loading procedures.

### Site-selective deposition of the metal cocatalyst

**Fig. 2:**
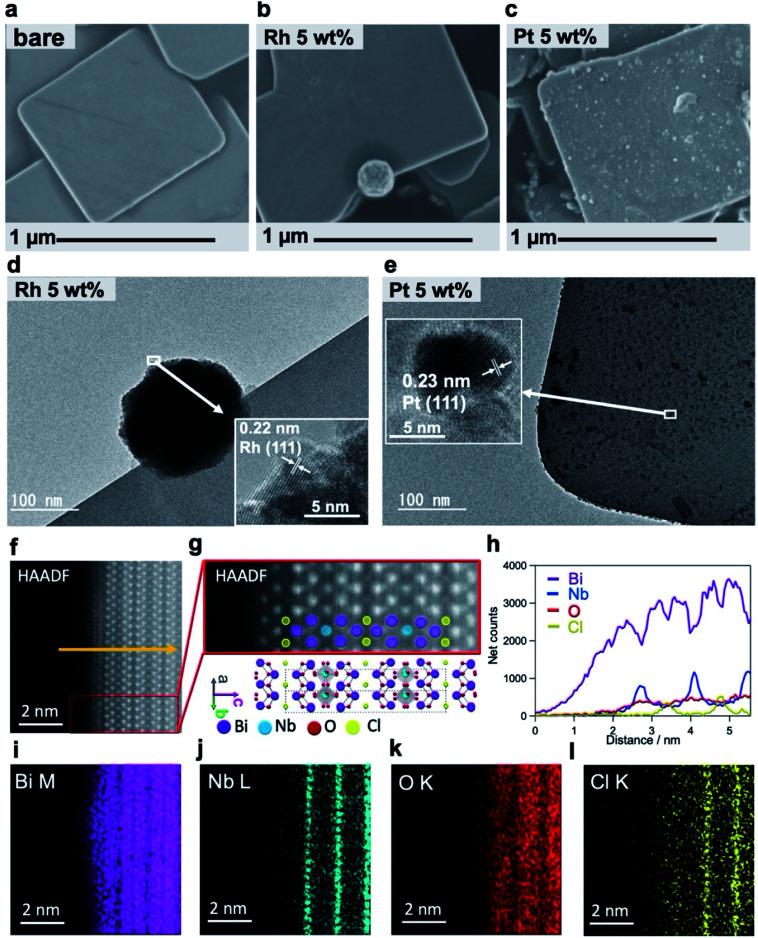
(a–c) SEM images of Bare-Bi_4_NbO_8_Cl (a) Rh-Bi_4_NbO_8_Cl (b) and Pt-Bi_4_NbO_8_Cl (c). (d and e) Bright-field TEM images of Rh-Bi_4_NbO_8_Cl (d) and Pt-Bi_4_NbO_8_Cl (e). The insets show HRTEM images of the cocatalysts. (f) HAADF-STEM image of a Bare-Bi_4_NbO_8_Cl nanoplate along the [110] direction. (g) Close-up view of the red rectangle in (f), together with the Bi_4_NbO_8_Cl crystal structure. (h–l) STEM-EDX line scan analysis (h) along the yellow arrow in (f) and atomic resolution elemental maps (i–l) for Bi (i) Nb (j) O (k) Cl (l).

We conducted detailed investigations using mainly electron microscopic tools (Fig. 2) to understand why Rh-Bi_4_NbO_8_Cl showed the excellent H_2_ evolution activity. Fig. 2a and S5† represent a typical SEM image of Bare-Bi_4_NbO_8_Cl synthesized by the flux method, which has nanoplate morphology with large aspect ratios; the typical lateral length and thickness are approximately 1 μm and 50 nm, respectively.^30^ The TEM/SAED pattern (Fig. S6†) indicated that the dominant facet is the (001) plane. HAADF-STEM images recorded along the [110] and [100] directions are shown in Fig. 2f, g and S7,† consistent with the reported crystal structure.^33^ Notably, the STEM/EDX line scan analysis and elemental maps suggest that the outermost layer is the [Bi_2_O_2_] layer (Fig. 2h–l and S7†).

A major difference between Rh-Bi_4_NbO_8_Cl and Pt-Bi_4_NbO_8_Cl is the location of the cocatalyst, as seen in SEM and HRTEM images; the Rh cocatalyst, deposited as agglomerations, was found only at the edges of the Bi_4_NbO_8_Cl nanoplate (Fig. 2b and d), whereas the Pt cocatalyst was observed as nanoparticulates dispersed mainly on the (001) facet (Fig. 2c and e). Absorption spectroscopy (Fig. S8†) ensured that precursors for the Rh and Pt cocatalysts, RhCl_6_^3−^ and PtCl_4_^2−^, were fully consumed during the PD process. The analysis of HRTEM (Fig. 2d and e) and XPS (Fig. S9†) confirmed that the Rh and Pt species were reduced to zero-valence by photogenerated electrons. The crystal phase and the valence state of Bi_4_NbO_8_Cl were not changed by the cocatalyst loading (Fig. S10†). For Rh-Bi_4_NbO_8_Cl, no Rh species were observed on the (001) facet (Fig. S11†), showing selective deposition at the edges. Direct photoreduction of Rh^3+^ was ruled out by a control experiment (Fig. S12†). We also verified that the p/n type, doping level, and zeta potentials were not affected by the Pt and Rh loading (Fig. S13†). The H_2_ evolution rate of Rh-Bi_4_NbO_8_Cl reached 222.4 μmol h^−1^ at 20 wt% loading (Fig. S14†), showing an apparent quantum efficiency (AQE) value of 2.6% at 405 nm (Table S1†); note that there was room for improvement as the Rh particles grew into aggregates (Fig. S15d–f, 16c and d†).

As in the case of Rh and Pt, photoreduction of Au^3+^ and Ag^+^ resulted in preferential deposition as elemental metals on the edges and dominant facet, respectively (Fig. S17†). Note that although Au was deposited at the edge as Rh, the Au-loaded Bi_4_NbO_8_Cl showed negligible activity (Fig. S1†), due to the much inferior HER catalytic activity of Au to Rh.^34^ The observed variation in metal deposition sites is unaccountable by the known facet-dependent redox mechanism in which reductive cocatalyst deposition should take place at fixed sites suitable for reduction, regardless of the metal species.^11^ A possible mechanism for the variation in the metal deposition site is described in Fig. S18,† where the reduction rate of each metal may be a key.

In general, dispersed nanoparticles with small overpotentials for target reactions are preferable as cocatalysts and can often enhance photocatalytic reactions.^35–37^ However, the contrasting H_2_ evolution activities of Pt-Bi_4_NbO_8_Cl and Rh-Bi_4_NbO_8_Cl suggest that the effect of the deposition site is pivotal, rather than the intrinsic catalytic capability of the cocatalyst, which is supported by the following five additional observations:

(1) The impregnation method produced Rh particles mainly dispersed on the (001) facet (with a minor fraction on the edge), which did not serve as an effective H_2_ evolution cocatalyst (Fig. S19†). (2) The Pt cocatalyst enhanced H_2_ evolution when loaded on the edges of Bi_4_NbO_8_Cl nanoplates. With increased loading (20 wt%), some Pt nanoparticles appeared on the edges of Pt-Bi_4_NbO_8_Cl (Fig. S15a–c, 16a and b†). The Pt-Bi_4_NbO_8_Cl sample with 20 wt% Pt exhibited a rate of H_2_ evolution of 65.9 μmol h^−1^ (Fig. S14†), which was greater than those of the samples with 1 wt% and 5 wt% Pt mainly on the (001) facet (0.3 and 2.4 μmol h^−1^). (3) When Pt was deposited on not only the (001) surface but also the edge at a high loading amount, the H_2_ evolution activity of Pt–Bi_4_NbO_8_Cl was not higher than that of Rh–Bi_4_NbO_8_Cl, regardless of whether the activity is plotted *versus* the loading amount in wt% or mol% (Fig. S14†), despite better catalytic activity for the HER of Pt than Rh. This result indicates that Pt on (001) affords a negative contribution to the H_2_ evolution. (4) When additional Pt (1 wt%) was deposited on the (001) facet of Rh-Bi_4_NbO_8_Cl with 5 wt% Rh, the photocatalytic activity deteriorated substantially (from 39.5 to 8.5 μmol h^−1^, Fig. S20†). (5) Rh-Bi_4_NbO_8_Cl showed a higher photocatalytic activity for not only water reduction but also water oxidation than Pt-Bi_4_NbO_8_Cl (Fig. S21a†), although the catalytic activity for IO_3_^−^ reduction of Rh is inferior to that of Pt (Fig. S21b and c†). These results clearly show that the deposition site, rather than the metal species, of the cocatalyst significantly affects the photocatalytic activity.

### Carrier flows in Bare-Bi_4_NbO_8_Cl

**Fig. 3:**
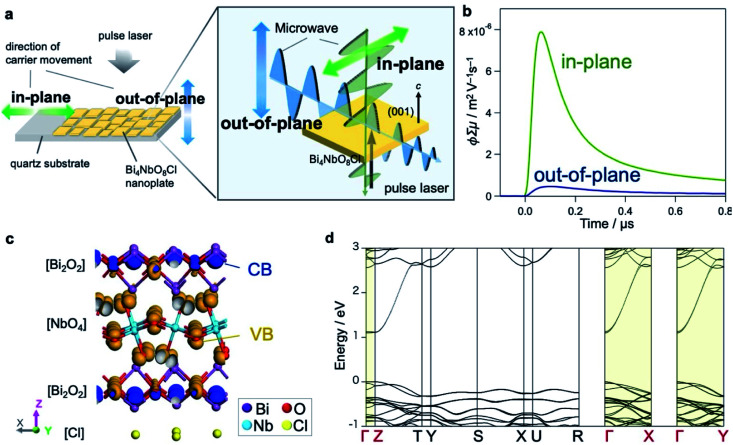
(a) Schematic illustration of the experimental setup for the anisotropic TRMC measurement. (b) Conductivity transients for Bare-Bi_4_NbO_8_Cl. Blue and green profiles are those observed along the in-plane and out-of-plane direction against the (001) plane corresponding to the blue and green arrows in (a), respectively. The vertical axis represents photoconductivity *φ*∑*μ*, where *φ* and ∑*μ* are the quantum efficiency of the charge carrier photogeneration and sum of the carrier mobilities, respectively. (c) Orbital distribution of the lower parts of the conduction band (blue) and the upper parts of the valence band (orange) of Bi_4_NbO_8_Cl estimated by DFT calculation. (d) Band structure of Bi_4_NbO_8_Cl, where both CB minimum and VB maximum are located at the *Γ* point.

We hereafter elucidate the dynamics of photoexcited carriers in Bi_4_NbO_8_Cl. Let us first discuss the results of time-resolved microwave conductivity (TRMC),^38,39^ which provides direct insight into the carrier flow in Bare-Bi_4_NbO_8_Cl. Of note, we adopted two measurement configurations (Fig. 3a) taking advantage of the morphology of Bi_4_NbO_8_Cl nanoplates; the anisotropy of carrier mobilities perpendicular and parallel to the (001) plane was evaluated by changing the direction of the incident microwave with respect to the nanoplates fixed to a substrate (Fig. S22†). The conductivity transients for the two settings are displayed in Fig. 3b, indicating an intense signal for the in-plane direction, with a maximum value of photoconductivity *φ*∑*μ* being 17 times higher. Provided that *φ* is constant (supported by the fact that the photoexcitation for carrier generation is fixed throughout the experiment), the TRMC results indicate that the Bi_4_NbO_8_Cl nanoplate exhibits a large anisotropy in the charge carrier flow, with greater mobility along the in-plane direction.

Theoretical investigations further support the anisotropic nature of the carrier flow in Bi_4_NbO_8_Cl. Fig. 3c depicts the orbital distribution estimated by DFT calculations, indicating that Bi-6p orbitals in the [Bi_2_O_2_] layer mainly contribute to the bottom of the conduction band (CB, blue) and O-2p orbitals in the [NbO_4_] layer to the top of the valence band (VB, orange). The band structure of Bi_4_NbO_8_Cl (Fig. 3d and S23†) shows that both the CB and VB have greater dispersion along the in-plane directions (*Γ*–*X* and *Γ*–*Y*) than the out-of-plane direction (*Γ*–*Z*), in common with layered materials (*e.g.*, PbBiO_2_Cl^24^ and Sr_2_TaO_3_N^40^). However, a closer look reveals that the CB is more dispersive than the VB, and the effective mass of electrons (0.25 *m*_0_) is smaller than that of holes (1.4 *m*_0_). Thus, electrons have in-plane mobility much higher than holes.

### Fate of the carriers

**Fig. 4:**
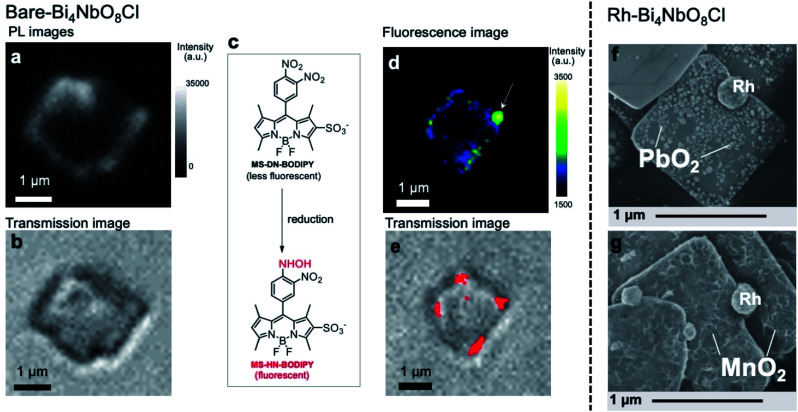
(a and b) PL (a) and transmission images (b) of a single Bare-Bi_4_NbO_8_Cl nanoplate immobilized on a cover glass in air under 405 nm laser irradiation. The PL intensity was integrated over all monitored wavelengths. (c) Reductive turn-on conversion of the fluorescent molecular probe used in single-molecule fluorescence imaging. (d and e) Fluorescence (d) and transmission (e) images of a single Bare-Bi_4_NbO_8_Cl nanoplate immobilized on a cover glass in an Ar-saturated aqueous methanol solution containing MS-DN-BODIPY (0.3 μM), wherein methanol served as a hole scavenger. For (d), the image was taken under 488 nm laser irradiation. The red dots in (e) represent fluorescence bursts observed during the 180 s irradiation. (f and g) SEM images of Rh-Bi_4_NbO_8_Cl after the photocatalytic deposition of PbO_2_ (f) and MnO_2_ (g).

The fate of photogenerated carriers in Bi_4_NbO_8_Cl was visualized by single-particle photoluminescence (PL) imaging,^41^ using recombination of the electrons and holes, or redox reaction products as probes. The PL image of a single Bare-Bi_4_NbO_8_Cl nanoplate excited by a 405 nm pulse laser (Fig. 4a and S24†) shows strong emission on the edges of the nanoplate when referring to a concurrent optical transmission image of the nanoplate (Fig. 4b). Herein we conclude that the optical waveguide effect does not play a chief role in the strong emission on the edge, because PL from the bulk and that from the edge possess lifetimes different from each other, and the waveguide effect is negligible when the particle is irradiated with a 532 nm laser that Bi_4_NbO_8_Cl does not absorb (Table S2 and Fig. S24†). Combined with the TRMC results, the single-particle PL imaging result of Bare-Bi_4_NbO_8_Cl indicates that both photogenerated carriers move in the in-plane direction, leading to recombination at the nanoplate edges.

We then performed two measurements in the presence of reactants to study the dynamics of either carrier, using the single-molecule fluorescence imaging technique for electrons^42,43^ and oxidative PD of metal oxides for holes. Fig. 4d shows the fluorescence image of a single Bare-Bi_4_NbO_8_Cl nanoplate excited at 488 nm in an Ar-saturated aqueous methanol solution containing an MS-DN-BODIPY molecular probe for electrons (Fig. 4c), wherein methanol served as a hole scavenger. Green and yellow spots showing intense fluorescence were observed mostly at the edge of the Bi_4_NbO_8_Cl nanoplate. These spots arose from the luminescent MS-HN-BODIPY converted from the non-luminescent MS-DN-BODIPY upon reduction by photogenerated electrons,^44^ as supported by the time evolution of fluorescence mapping (Fig. S25†). Several fluorescence bursts were detected over 180 s of photoirradiation, which are displayed in Fig. 4e as red spots overlaid with a concurrent optical transmission image of the nanoplate, with the reproducibility checked on other nanoplates (Fig. S26†). Overall, the single-molecule fluorescence experiments show that photoexcited electrons predominantly move along the in-plane direction and accumulate at the nanoplate edge.

Turning to the holes, we conducted the photocatalytic oxidation of Pb^2+^ and Mn^2+^ in an oxygen-saturated aqueous solution. Here, the metal ions are oxidized by photogenerated holes to the corresponding metal oxides (PbO_2_ and MnO_2_) deposited on the photocatalyst surface, thereby acting as probes for the oxidation sites of photocatalysts, where Pb^2+^ and Mn^2+^ have often been employed.^11^ On the other hand, photoexcited electrons are trapped by electron acceptors (O_2_ and/or H_2_O). For Bare-Bi_4_NbO_8_Cl nanoplates, negligible metal oxide deposition was observed under visible light irradiation (*λ* > 400 nm) (Fig. S27†), which suggests that holes recombine with electrons before participating in the oxidation. In contrast, Rh-Bi_4_NbO_8_Cl experienced PbO_2_ and MnO_2_ deposition (Fig. S28–S31†); a number of particles and sponge-like deposits, in the cases of Pb^2+^ and Mn^2+^, respectively, were scattered on the (001) facet (Fig. 4f and g). The result for Rh-Bi_4_NbO_8_Cl is particularly important as it indicates that photogenerated holes are allowed to migrate along the out-of-plane direction and reach the (001) surface, despite the dispersionless VB structure along this direction. Together with the hydrogen evolution experiments (Fig. 1b), we conclude that the carrier dynamics of Rh-Bi_4_NbO_8_Cl is characterized by a unique charge separation, where electrons and holes are spatially separated and migrate, respectively, toward Rh at the nanoplate edge and toward the basal plane, triggering their respective redox reactions.

### Carrier decay dynamics

**Fig. 5:**
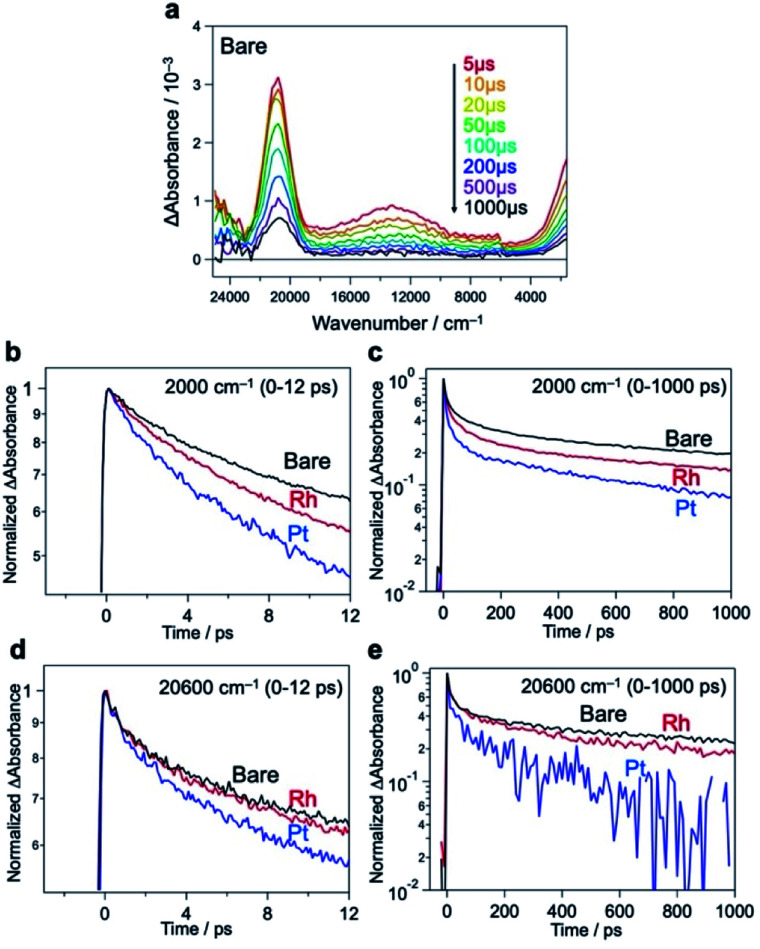
(a) Microsecond TRAS of Bare-Bi_4_NbO_8_Cl measured in the vacuum. The sample was excited by 355 nm laser pulses (6 ns duration, 0.5 mJ, 5 Hz). (b–e), Femtosecond decay profiles of transient absorptions at 2000 cm^−1^ (b and c) and 20 600 cm^−1^ (d and e) for Bare-Bi_4_NbO_8_Cl, Rh-Bi_4_NbO_8_Cl, and Pt-Bi_4_NbO_8_Cl. The samples were excited by 355 nm laser pulses (90 fs duration, 6 μJ, 500 Hz) in air for the several pico-second (ps) region.

We further compared the charge carrier decay profiles using time-resolved absorption spectroscopy (TRAS), as used for semiconductor photocatalysts.^45,46^ A microsecond transient spectrum for Bare-Bi_4_NbO_8_Cl, when excited by a 355 nm laser pulse (Fig. 5a), shows three characteristic absorption peaks at 2000 cm^−1^, 13 000 cm^−1^, and 20 600 cm^−1^. Referring to previous results (*e.g.*, TiO_2_,^47–49^ α-Fe_2_O_3_ ^50^ and LaTiO_2_N^45^), the first absorption is attributed to photogenerated free (or shallowly trapped) electrons in the CB,^51,52^ the second one to the excitation of trapped electrons from the mid-gap state to the CB^46^ (derived from halogen defects, see Fig. S32†), and the third one to the photogenerated holes. The initial decay profiles after photoexcitation were examined using femtosecond TARS, focusing on the absorbance changes at 2000 and 20 600 cm^−1^ (Fig. 5b–e). The decay of free electrons at 2000 cm^−1^ was accelerated by the loading of Rh or Pt compared to Bare-Bi_4_NbO_8_Cl (Fig. 5b and c); especially, Pt-Bi_4_NbO_8_Cl decayed more rapidly than Rh-Bi_4_NbO_8_Cl. Regarding hole absorption at 20 600 cm^−1^ (Fig. 5d and e), the Pt cocatalyst accelerated a decline in the absorption peak, while the decay acceleration was less prominent in Rh-Bi_4_NbO_8_Cl. These observations suggest that Pt on the (001) facet traps both electrons and holes, while Rh at the edge captures only electrons, leaving holes in the bulk of Bi_4_NbO_8_Cl on picosecond timescales.

### Carrier flow engineering

**Fig. 6:**
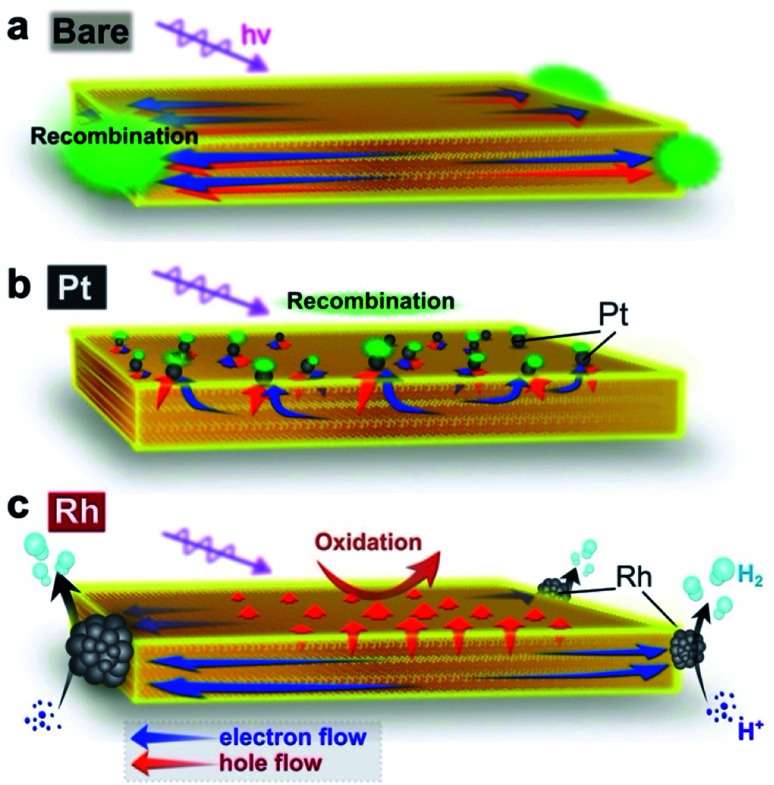
(a–c) Suggested major carrier flow in Bare-Bi_4_NbO_8_Cl (a), Pt-Bi_4_NbO_8_Cl (b) and Rh-Bi_4_NbO_8_Cl (c). In Bare-Bi_4_NbO_8_Cl, both electrons and holes are prone to move in the in-plane direction according to their band dispersions, resulting in recombination at the nanoplate edges. In Pt-Bi_4_NbO_8_Cl, both carriers are collected by the Pt cocatalyst based on the band bending mechanism, leading again to recombination (Fig. 6b). However, the edge-deposited Rh cocatalyst in Rh-Bi_4_NbO_8_Cl can collect photogenerated “light” electrons predominantly, while “heavy” holes left in the photocatalyst hop towards the (001) facet thanks to the anisotropic crystalline shape (approximately 50 nm in thickness). The orthogonal carrier flow leads to the high photocatalytic hydrogen activity.

Fig. 6 illustrates the suggested carrier flow dynamics and charge separation of the three Bi_4_NbO_8_Cl nanoplate samples. The thorough and systematic study described above allows us to reveal how the deposition site of the cocatalyst affects the direction of the carrier flow in the bulk and influences the H_2_ evolution photocatalytic activity. Hereafter, the carrier flow in each material is discussed from the viewpoints of solid-state chemistry, solid state physics, semiconductor engineering, and catalysis science.

In Bare-Bi_4_NbO_8_Cl, photogenerated carriers (both electrons and holes) are likely to travel in-plane, as expected from the layered crystal structure and band dispersion. More precisely, the in-plane dispersion of the conduction band is greater than that of the valence band (Fig. 3d), suggesting that electrons are lighter than the holes and can migrate faster along this direction. However, since the bare surface of Bi_4_NbO_8_Cl has no catalytic site for H^+^ reduction, the lighter electrons cannot flow out of the nanoplate. As a result, the electrons are trapped by the holes that subsequently reach the nanoplate edges, causing recombination, as confirmed by PL imaging (Fig. 4).

Once Rh is loaded on the nanoplate edges (Rh-Bi_4_NbO_8_Cl), the situation changes drastically. Herein, the “light” electrons, which arrive at the edge first, can be extracted from the nanoplate by Rh and then used in the reduction of H^+^ (or H_2_O), instead of recombining with the holes, as demonstrated by TRAS (Fig. 5). With the electrons being consumed, the photo-generated “heavy” holes must move to the surface of the nanoplate to react with methanol in order to satisfy charge neutrality in the bulk Bi_4_NbO_8_Cl. Of importance here is that our Bi_4_NbO_8_Cl crystals are in the form of nanoplates with a thickness of about 50 nm, and this forces the holes to hop towards the nearest (001) facet (Fig. 6c) against the dispersionless out-of-plane VB. The resultant orthogonal carrier flow realizes the spatial separation of photoexcited electrons and holes, greatly enhancing the photocatalytic activity.

In Pt-Bi_4_NbO_8_Cl, Pt captures both electrons and holes, in contrast to what the band dispersion of Bi_4_NbO_8_Cl tells us (Fig. 6b). Here, changes in the flows of both carriers are accounted for by band bending. In general, when novel metals such as Pt are brought into contact with an n-type semiconductor, upward band bending and Schottky barrier are formed in the space charge layer.^53^ In fact, such upward band bending was reported to allow photogenerated holes to migrate from n-type photocatalysts to Pt.^54–56^ In Pt-Bi_4_NbO_8_Cl, Pt nanoparticles are highly dispersed on the basal plane (*i.e.*, (001) facet) of the nanoplate (Fig. 2c). Considering the fact that the typical thickness of the space charge layer is a few tens of nanometers,^57^ the thickness of about 50 nm in the present nanoplates means that the scattered Pt and n-type Bi_4_NbO_8_Cl junctions give effective upward band bending over the entire crystal, allowing the photogenerated holes to move into Pt. On the other hand, the photogenerated electrons are prone to move in the in-plane direction toward the edge of Bi_4_NbO_8_Cl due to the high mobility along the in-plane direction. However, in Pt-Bi_4_NbO_8_Cl, they cannot be consumed at the edge, because of the inertness to H^+^ reduction in the absence of a cocatalyst there. As a result, electrons are eventually captured by Pt on the (001) plane, followed by recombination with holes therein, as shown by TRAS (Fig. 5). The electron transfer mechanism from Bi_4_NbO_8_Cl to Pt might be tunneling through the Schottky barrier,^58–60^ where the high dispersity of Pt on the (001) facet should increase the probability of electron transfer to Pt *via* tunneling. Note that the preceding hole transfer from Bi_4_NbO_8_Cl to Pt reduces the band bending and barrier height, which may make the subsequent electron transfer easier. In addition, the requirement for charge neutrality in the bulk Bi_4_NbO_8_Cl after the hole transfer to Pt may also be at play in the electron transfer to Pt and subsequent recombination with the holes. Another possible case is that, at the edge, electrons are recombined with holes that are not captured by Pt as in Bare-Bi_4_NbO_8_Cl.

Recall that previous studies on photocatalytic systems relied on facet engineering for efficient charge separation, specifically, in TiO_2_,^10^ BiVO_4_,^11^ SrTiO_3_ ^12^ and Bi_2_MoO_6_,^13^ where the photooxidation and reduction sites are separated inherently on different facets. For example, in BiVO_4_,^11^ photo-oxidation and photo-reduction take place preferentially on the (110) and (010) facets, respectively, which is associated with the VBM/CBM levels and the energy of each facet surface. This means that the direction of the carrier flow is determined by the inherent properties of photocatalysts themselves. The choice of the facet plane is a parameter to control. However, there are difficulties and uncertainties in exposing the desired facet experimentally. The present study proposes another strategy for the efficient charge separation: site-specific metal loading manipulates the carrier flow of layered Bi_4_NbO_8_Cl; the intrinsic but unfavorable parallel carrier flow was changed into the orthogonal one.

## Conclusions

In summary, we successfully manipulated the carrier flow in a layered Bi_4_NbO_8_Cl nanoplate suffering from charge recombination by Rh deposition at the edge, which leads to the spatial charge separation and significantly improves the photocatalytic activity. In conjunction with differences in the in-plane dispersion between the conduction and valence bands, the site-selective deposition allows the Rh cocatalyst to extract photoexcited light electrons efficiently from the photocatalyst, which then forces the remaining heavy holes to hop perpendicular to the plane with the aid of anisotropic crystal geometry. We believe the present strategy is applicable not only to other Sillén(-Aurivillius) layered oxyhalide series,^31,61,62^ but also to a variety of semiconductor photocatalysts including mixed-anion compounds with narrow band gaps;^5,9,63^ above all, those considered to be useless or inefficient as photocatalysts can be transformed into effective ones by metal-species deposition on appropriate sites using the knowledge on band structures. The present work provides a deeper understanding on, and a new insight for charge separation in semiconductor photocatalysts.

## Experimental section

### Materials

CsCl (99.0%), NaCl (99.5%), Bi_2_O_3_ (99.99%), BiOCl (99.5%), Nb_2_O_5_ (99.9%), H_2_PtCl_6_ (99.9%), CH_3_OH (99.8%), Pb(NO_3_)_2_ (99.9%), and MnSO_4_·5H_2_O (99.9%) were purchased from FUJIFILM Wako Pure Chemical Corporation. Rh(NO_3_)_3_ and Na_3_RhCl_6_ were purchased from Kanto Chemical Corporation. Sodium 2-sulfonate-1,3,5,7-tetramethyl-8-(3,4-dinitrophenyl)-4,4-difluoro-4-bora-3a,4a-diaza-s-indacene (MS-DN-BODIPY) was synthesized according to the literature. Water was purified using a Milli-Q purification system (Direct-Q UV S.).

### Preparation of Bi_4_NbO_8_Cl nanoplates

Bi_4_NbO_8_Cl nanoplates were synthesized *via* the flux method according to our previous report.^30^ A eutectic mixture (65 : 35) of CsCl and NaCl was used as a flux. The flux was mixed with a stoichiometric mixture of Bi_2_O_3_, BiOCl, and Nb_2_O_5_ at a solute concentration of 1 mol%. The total mass was set to be 25 g. The mixture was placed in an alumina crucible with a capacity of 30 cm^3^, and then heated at a rate of 50 °C h^−1^ to 650 °C, being held at the final temperature for 10 h. After the natural cooling under ambient conditions, the product was washed thoroughly with deionized water, collected by filtration, and air-dried.

### Cocatalyst loading

Rh and Pt and other cocatalysts (Ru, Ir, and Pd) were loaded on Bi_4_NbO_8_Cl by photo-deposition (PD) and impregnation. In the PD process, metal cations in the precursors are reduced by photogenerated electrons and deposited on photocatalysts. The following precursors were employed: H_2_PtCl_6_, RuCl_3_, Na_3_IrCl_6_, H_2_PdCl_4_ and Na_3_RhCl_6_. In a typical procedure of photo-deposition, 0.2 g of Bi_4_NbO_8_Cl powder and a calculated amount of metal precursor (0.5–20 wt% for Pt and Rh, 0.5 wt% for others) were mixed in 250 mL of 20 vol% methanol aqueous solution. The suspension was then irradiated with visible light (*λ* > 400 nm) for 5–12 h through a cutoff filter from a 300 W Xe-arc lamp with continuous stirring in a Pyrex reaction vessel connected to a closed circulation system in an Ar atmosphere at around 298 K.

On the other hand, in the impregnation method, the deposition process is initiated by the “forced” adsorption of metal cations onto photocatalyst surfaces by solvent evaporation, followed by thermal reduction.^5^ First, 0.2 g of Bi_4_NbO_8_Cl powder was immersed in an aqueous solution containing each precursor (0.5 wt%). In the case of 5 wt% of Rh deposition by the impregnation method, Rh(NO_3_)_3_ was used. The suspension was evaporated under constant stirring until complete dryness was reached, followed by heating in an H_2_ flow at 200 °C for 30 min.

### Characterization

Scanning electron microscopy (SEM) images were taken using a Zeiss Nvision 40 microscope. High-angle annular dark-field scanning transmission electron microscopy (HAADF-STEM) and annular bright-field scanning transmission electron microscopy (ABF-STEM) images were collected using a JEM-ARM200CF (JEOL Ltd, Tokyo, Japan) operating at an accelerating voltage of 200 kV and equipped with a cold field emission gun and a Cs corrector to observe atomic columns of Bi_4_NbO_8_Cl. Elemental analysis was carried out using a JEM-ARM200CF equipped with energy dispersive X-ray spectroscopy (EDX). The samples were prepared by grinding the material and depositing a few drops of the suspension onto a holey copper grid covered with a thin carbon film. Transmission electron microscopy (TEM) was carried out using a JEOL JEM-2100F microscope. X-ray photoelectron spectroscopy (XPS) measurements were performed with an ULVAC-PHI 5500MT system.

### Photocatalytic reaction

Photocatalytic reactions were performed in a gas closed-circulation system. The photocatalyst powder (0.1 g) was dispersed in a methanol aqueous solution (20 vol%, 100 mL) in a Pyrex top-window cell. The photocatalyst was irradiated with UV and visible light (*λ* > 300 nm) or visible light (*λ* > 400 nm) through a cutoff filter from a 300 W Xe-arc lamp. The quantity of the evolved gas was determined using an online gas chromatograph (thermal conductivity detector; molecular sieve 5 Å column packing; Ar carrier gas). The apparent quantum efficiency (AQE) was evaluated using a 405 nm monochromatic LED light source (ASAHI SPECTRA, CL-1501).

### Single-molecule fluorescence imaging

To obtain isolated Bi_4_NbO_8_Cl particles, a well-dispersed methanol suspension of Bi_4_NbO_8_Cl in low concentration was spin-coated onto a cleaned cover glass. The particle-coated cover glass was annealed at 363 K for 30 min to immobilize the particles on the glass surface, and then placed in a chamber filled with an Ar-saturated aqueous methanol solution of MS-DN-BODIPY (1 μM). A 488 nm CW laser (OBIS 488LX, Coherent; 10 mW cm^−2^) passing through an objective lens (CFI Plan Apo λ100 × H, Nikon; NA 1.45) after reflection by a dichroic mirror (Di02-R488, Semrock) was reflected completely at the cover glass–solution interface to generate an evanescent field, which made it possible to detect the fluorescent products selectively on the bottom surface of the crystal. The emission from the sample was collected by the same objective lens, after which it was magnified by a 1.5× built-in magnification changer, and passed through a band-pass filter (FF01-535/50, Semrock) to remove undesired scattered light. The emission images were recorded using an electron-multiplying charge-coupled device (EMCCD) camera (Evolve 512, Roper Scientific) using Micro-Manager (https://www.micro-manager.org/). All experimental data were obtained at room temperature.

### Single-particle PL imaging

PL microscopy measurement was also conducted based on a Nikon Ti-E inverted fluorescence microscope. For wide-field microscopy, the 405 nm CW laser (OBIS 405LX, Coherent; 30 mW cm^−2^) was used to excite the Bi_4_NbO_8_Cl. The emission images were recorded on an EMCCD at a rate of 30 frames s^−1^. A suitable dichroic mirror (Di02-R488, Semrock) and a long-pass filter (BLP01-488R, Semrock) were used to improve the signal-to-noise ratio. For confocal microscopy, the 405 nm pulsed diode laser (Advanced Laser Diode System, PiL040X; 45 ps FWHM, 1 MHz repetition rate) was used to excite the samples. The emitted photons were passed through a 100 μm pinhole and then directed onto a single-photon avalanche diode (Micro Photon Devices, SPD-050). The signals from the detector were sent to a time-correlated single photon counting module (Becker & Hickl, SPC-130EM) for further analysis. A dichroic mirror (Semrock, Di02-R405) and a longpass filter (Semrock, BLP01-458R) were used to remove the scattering from the excitation light. PL spectra were obtained by directing the emission into an imaging spectrograph (SOL instruments, MS3504i) equipped with a CCD camera (Andor, DU416A-LDC-DD) through a slit. All experimental data were obtained at room temperature in air. The data were analyzed using the open source image software ImageJ (http://rsb.info.nih.gov/ij/) and Origin 2015 (Origin-Lab).

### Photocatalytic deposition of PbO_2_ and MnO_2_

Photo-deposition of PbO_2_ or MnO_2_ was conducted with Pb(NO_3_)_2_ or MnSO_4_. Photocatalyst powders (20 mg) were dispersed in 10 mL of an aqueous Pb(NO_3_)_2_ or MnSO_4_ solution (5 wt% as Pb or Mn) in a test tube. After the suspension was purged with O_2_ gas for 30 min, visible light (*λ* > 400 nm) was irradiated using a 300 W Xe-arc lamp. After the 3 h irradiation, the suspension was filtered, washed with deionized water and dried at room temperature.

### TRMC measurements

X-band microwave (∼9.1 GHz) and third harmonic generation (THG; 355 nm) of a Nd:YAG laser (Continuum Inc., Surelite II, 5–8 ns pulse duration, 10 Hz) were used as the probe and band-gap excitation (4.6 × 10^15^ photons per cm^2^ per pulse), respectively. A powdery Bi_4_NbO_8_Cl sample was fixed onto a quartz substrate using optically clear adhesive tape that does not interfere with any TRMC signal. The photoconductivity Δ*σ* was calculated using the following formula: Δ*σ* = Δ*P*_r_/(*AP*_r_), where Δ*P*_r_, *A*, and *P*_r_ are the transient power change of the reflected microwave, the sensitivity factor, and the power of reflected microwave, respectively. The obtained Δ*σ* values were then converted into the product of the quantum yield (*φ*) and the sum of the carrier mobilities (∑*μ* = *μ*_+_ + *μ*_−_) using the following formula: *φ*∑*μ* = Δ*σ*(*eI*_0_*F*_light_)^−1^, in which *e* and *F*_light_ are the unit charge of a single electron and a correction (or filling) factor, respectively. All TRMC measurements were performed in an ambient atmosphere at 25 °C.

### TRAS measurements

A set of custom-built TRAS spectrometers was employed as described previously.^45^ In the femtosecond to nanosecond regions, experiments were performed using a conventional pump–probe method based on a Ti:sapphire laser system (Spectra Physics, Solstice & TOPAS Prime; duration, 90 fs; repetition rate, 1 kHz). In this experiment, a 355 nm laser pulse was used as the pump pulse. The experiments were performed in air to prevent heating of the sample and to minimize the accumulation of electrons in the photocatalyst due to the high-frequency pump pulse irradiation (500 Hz). In the microsecond to seconds region, transient absorption spectra were measured from 25 000 to 1 000 cm^−1^. 355 nm light (Continuum, Surelite-II, 6 ns, 355 nm, repetition rate of 5–0.01 Hz) was used as the pump pulse. The spectra were obtained at intervals of 200 cm^−1^ and averaged over 300 scans per spectrum. The measurements were performed under vacuum at room temperature. The powder photocatalyst was fixed on a CaF_2_ plate with a density of ∼1 mg cm^−2^, and the sample plate obtained was placed in a stainless steel cell. For fair comparison with PD-Rh-Bi_4_NbO_8_Cl and PD-Pt-Bi_4_NbO_8_Cl, Bare-Bi_4_NbO_8_Cl was irradiated using visible light for 5 h in a MeOH solution prior to use.

### Density functional theory calculation

The band structure calculation of Bi_4_NbO_8_Cl was performed within the framework of Density Functional Theory (DFT) using a plane-wave pseudopotential method as implemented in the Cambridge Serial Total Energy Package (CASTEP) code of BIOVIA's Material Studio 2019.^64^ The interaction between the ionic core and valence electrons was treated with the OTFG ultra-soft pseudopotential using the scalar relativistic Kolling-Harmon approximation. The non-scalar relativistic effects and spin–orbital coupling (SOC) were not considered. The Perdew–Burke–Ernzerhof (PBE) function of generalized gradient approximation (GGA) was employed as the exchange–correlation functional. A plane wave basis set with an energy cut off of 630 eV and the Monkhorst-pack 3 × 3 × 1 *k*-point mesh was used. The minimization algorithm of Broyden–Fletcher–Goldfarb–Shanno (BFGS) was employed for geometry optimizations with a total energy convergence tolerance of 10^−6^ eV per atom. The separation between *k*-points in the band structure calculations was 0.001 Å. Other convergence parameters are as follows: a self-consistent field tolerance of 1 × 10^−5^ eV per atom, a maximum stress of 0.05 GPa, and the maximum ionic displacement of 1 × 10^−3^ Å.

Effective mass m* was calculated according to the obtained band structure. m* is defined by the following equation:1
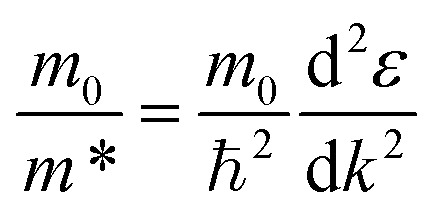
where *m*_0_ is the free electron mass, *k* is the reciprocal lattice vector in the direction of interest, and d^2^*ε*/d*k*^2^ is the curvature of the band at a maximum or a minimum, respectively. Assuming the band around their minima/maxima to be parabolic, we estimate the curvature of the band using the finite differences approximation:2
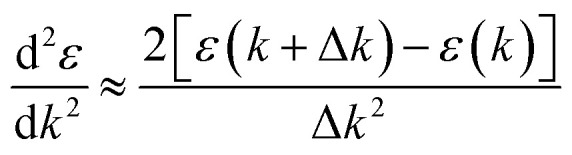
where Δ*k* = 0.05 Å.

## Author contributions

Kanta Ogawa: conceptualization, investigation, methodology, writing – original draft. Ryota Sakamoto: conceptualization, writing – review & editing. Chengchao Zhong: writing – review & editing. Hajime Suzuki: writing – review & editing. Kosaku Kato: investigation. Osamu Tomita: methodology. Kouichi Nakashima: resources. Akira Yamakata: resources, data curation. Takashi Tachikawa: resources, investigation, data curation, writing – review & editing. Akinori Saeki: resources, data curation. Hiroshi Kageyama: supervision, writing – review & editing. Ryu Abe: resources, supervision, writing – review & editing.

## Conflicts of interest

There are no conflicts to declare.

## Supplementary Material

SC-013-D1SC06054F-s001
