# Supplementary material for: Manipulation of charge carrier flow in Bi4NbO8Cl nanoplate photocatalyst with metal loading
Source: Chem Sci. 2022 Jan 24;13(11):3118–28. doi: 10.1039/d1sc06054f (PMC8926197; doi:10.1039/d1sc06054f)
Supplement: SC-013-D1SC06054F-s001 [file SC-013-D1SC06054F-s001.pdf]

Electronic Supplementary Material (ESI) for Chemical Science.
This journal is © The Royal Society of Chemistry 2022

Supplementary Information

Manipulation of charge carrier flow in Bi_4NbO_8Cl nanoplate photocatalyst with metal loading

Kanta Ogawa[1], Ryota Sakamoto[1], Chengchao Zhong[1], Hajime Suzuki[1], Kosaku Kato[2], Osamu Tomita[1], Kouichi Nakashima[3], Akira Yamakata[2], Takashi Tachikawa[4], Akinori Saeki[5], Hiroshi Kageyama[1,*], Ryu Abe[1,*]

[1] Department of Energy and Hydrocarbon Chemistry, Graduate School of Engineering, Kyoto University, Nishikyo-ku, Kyoto 615-8510, Japan

[2] Graduate School of Engineering, Toyota Technological Institute, 2-12-1 Hisakata, Tempaku, Nagoya 468-8511, Japan

[3] Department of Materials Science and Engineering, College of Engineering, Ibaraki University, 4-12-1, Nakanarusawa, Hitachi, Ibaraki 316-8511, Japan

[4] Molecular Photoscience Research Center, Kobe University, 1-1 Rokkodai-cho, Kobe 657-8501, Japan

[5] Department of Applied Chemistry, Graduate School of Engineering, Osaka University, 2-1 Yamadaoka, Suita, Osaka 565-0871, Japan

* Corresponding Author: kage@scl.kyoto-u.ac.jp, ryu-abe@scl.kyoto-u.ac.jp

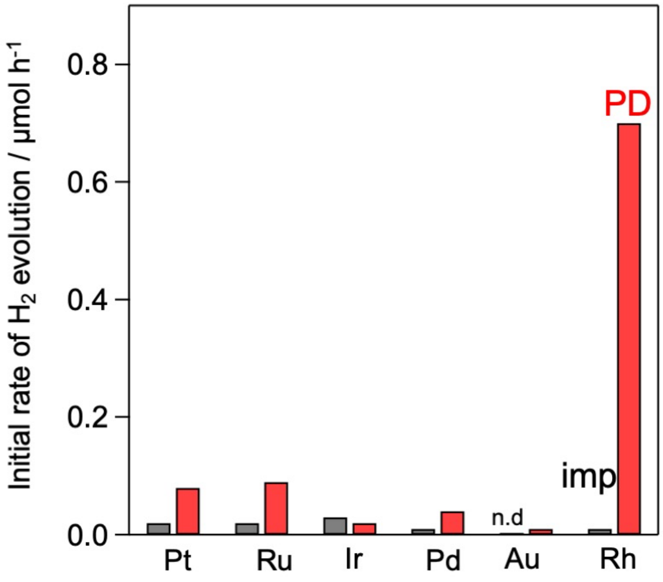

Fig. S1| Effect of cocatalyst loading on H_2 evolution activity over Bi_4NbO_8Cl photocatalyst. Various metal cocatalysts (0.5 wt% calculated as metal) were loaded by the photodeposition (PD) or impregnation (imp) method.

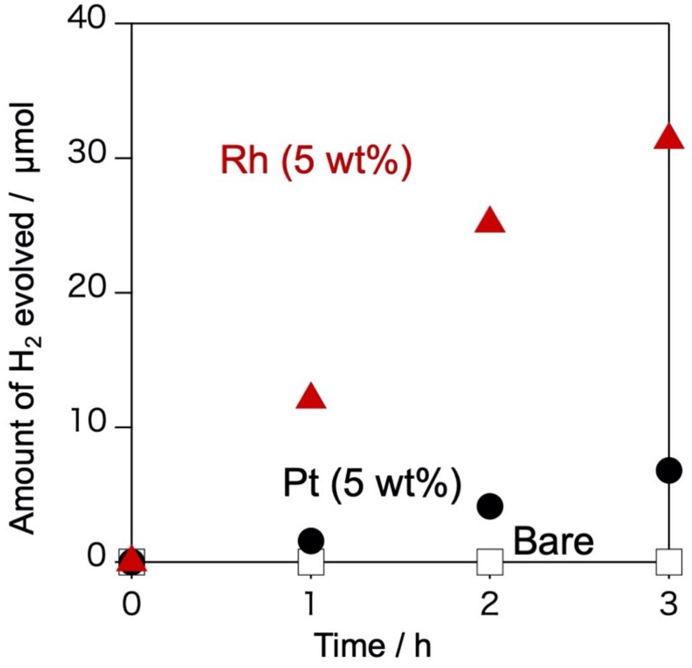

Fig. S2| Visible-light H_2 evolution activity. Time courses of H_2 evolution over Bare-Bi_4NbO_8Cl (squares), Rh-Bi_4NbO_8Cl (triangles) and Pt-Bi_4NbO_8Cl (circles) in a methanol aqueous solution (20 vol%) under visible-light irradiation ($\lambda > 400$ nm).

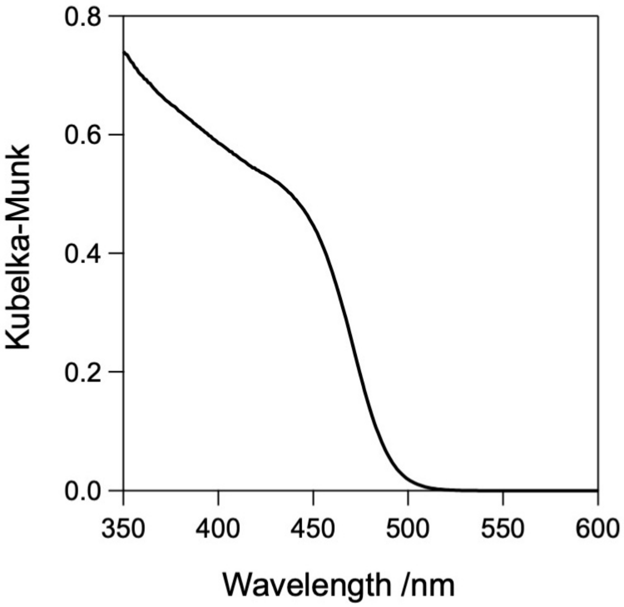

Fig. S3| Diffuse reflectance spectra of Bi_4NbO_8Cl

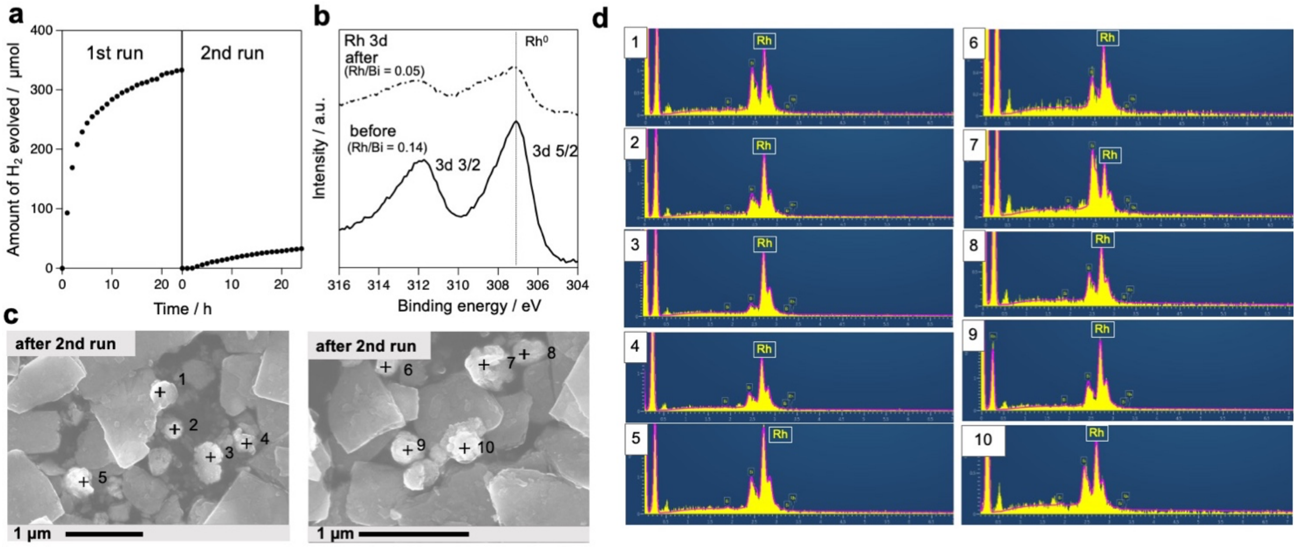

Fig. S4| Stability of photocatalytic H_2 evolution. a, Repeatability test for photocatalytic H_2 evolution using **Rh-Bi_4NbO_8Cl** (20 wt% Rh) in a methanol aqueous solution (20 vol%) under visible light irradiation ($\lambda > 400$ nm). **b,** XP spectra of Rh 3d regions of **Rh-Bi_4NbO_8Cl** before and after the catalytic reaction in **a**. **c, d,** SEM images and EDX analysis for **Rh-Bi_4NbO_8Cl** after the photocatalytic H_2 evolution reaction. The H_2 evolution activity of **Rh-Bi_4NbO_8Cl** was deteriorated after 24 h operation. This is due to the detachment of Rh from the photocatalyst during the catalytic process, where the photocatalyst suspension was illuminated upon continuous stirring. After the photocatalytic reaction, XPS showed a decrease in the

relative intensity of Rh with respect to Bi. Herein, the photocatalytic particles were collected by filtration with a PTFE membrane filter having 0.2 μm pore size (Omnipore). Therefore, the detached Rh nanoparticles smaller than 0.2 μm were not be collected, which resulted in the decreased intensity of Rh with respect to Bi after the reaction. Some of the Rh particles peeled off from the Bi_4NbO_8Cl nanoplate were observed also in SEM images. Note that such cocatalyst detachment is often problematic in various photocatalytic systems.[1–3] In the present case, the Rh cocatalyst is aggregated largely, which could promote the mechanical detachment upon stirring.

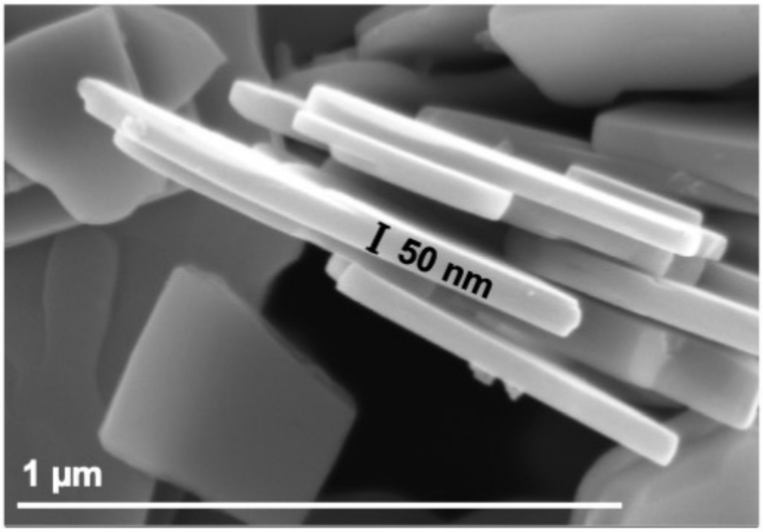

Fig. S5| SEM images of Bare-Bi_4NbO_8Cl

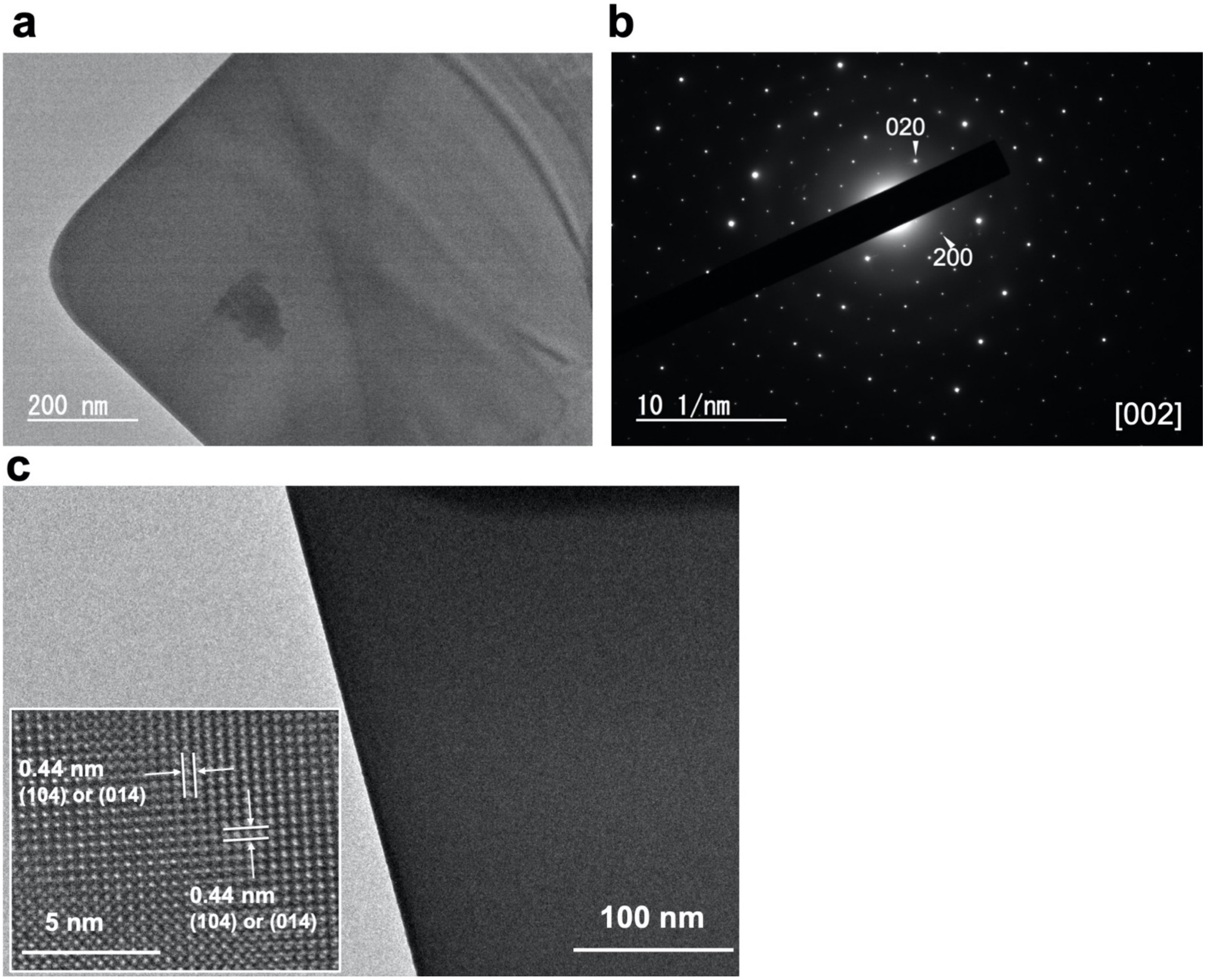

Fig. S6| Assignment of the basal plane of the Bi_4NbO_8Cl nanoplate. Bright-field TEM images (**a, c**) and TEM/SAED (**b**) for the Bi_4NbO_8Cl nanoplate. **a** and **b** are reprinted with permission from ref. [4]. Copyright 2019 American Chemical Society. These results show that the basal plane is assignable to the (001) plane and a high quality of crystals with little stacking fault.

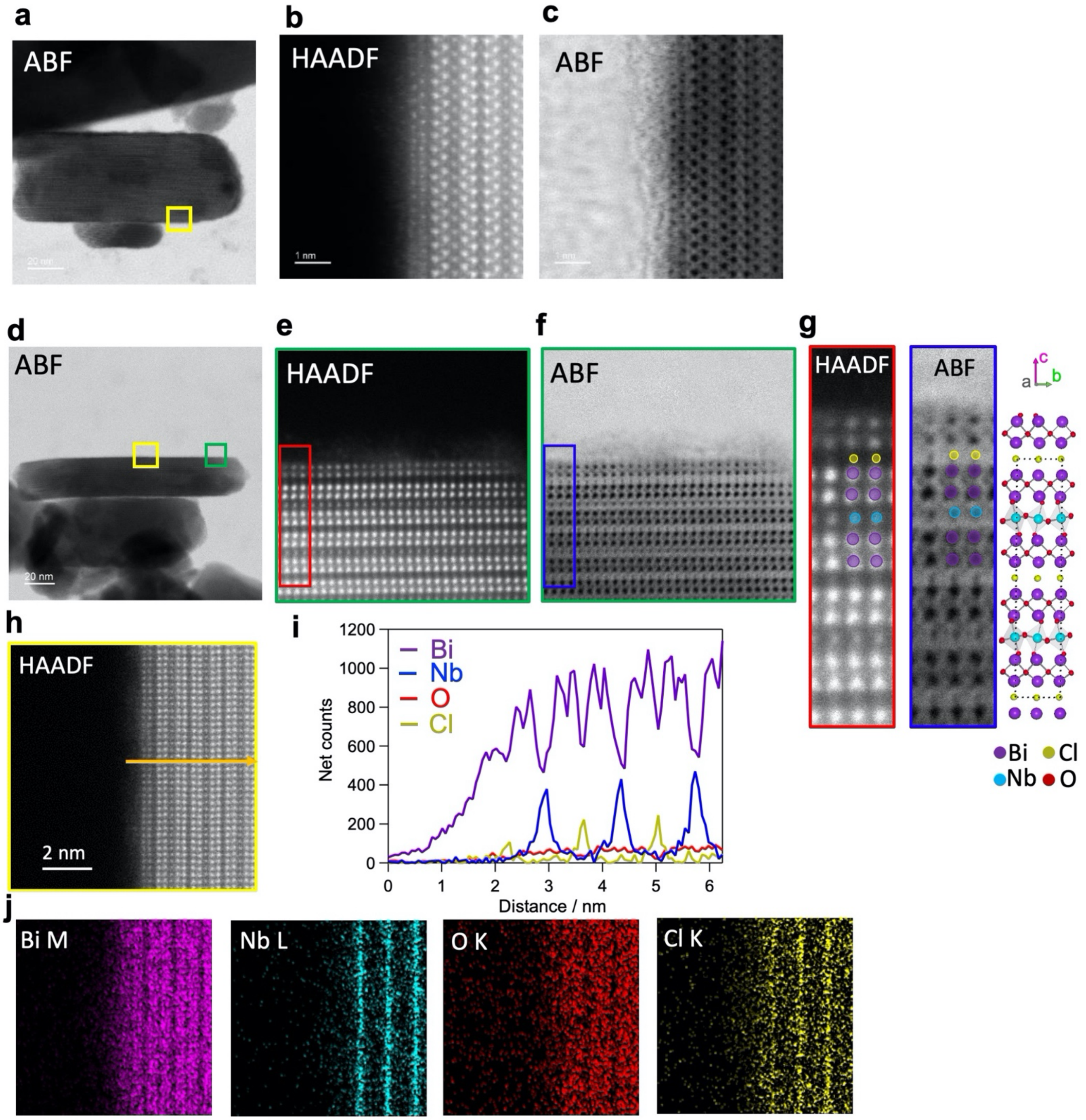

Fig. S7| Crystal structure and the outermost layer of the Bi_4NbO_8Cl nanoplate. a-c, HAADF (b) and ABF (**a, c**) STEM images for the same Bi_4NbO_8Cl particle shown in Fig. 2. **b, c** Zoomed view of the yellow box in **a**. **d,** ABF-STEM image of another Bi_4NbO_8Cl nanoplate along the [100] direction. **e, f,** Zoomed view of the green box in **d**. **g,** Close-up in the red (HAADF) and blue (ABF) rectangles in **e** and **f**, respectively, along with the corresponding Bi_4NbO_8Cl crystal structure. **h,** Zoomed view of the yellow

box in d. **i, j,** Atomic resolution STEM-EDX line analysis (**i**) along the yellow arrow in **h** and elemental maps (j). These results are consistent with the chemical composition and crystal structure of Bi_4NbO_8Cl and also show that the outermost layer comprises of the $[Bi_2O_2]$ layer.

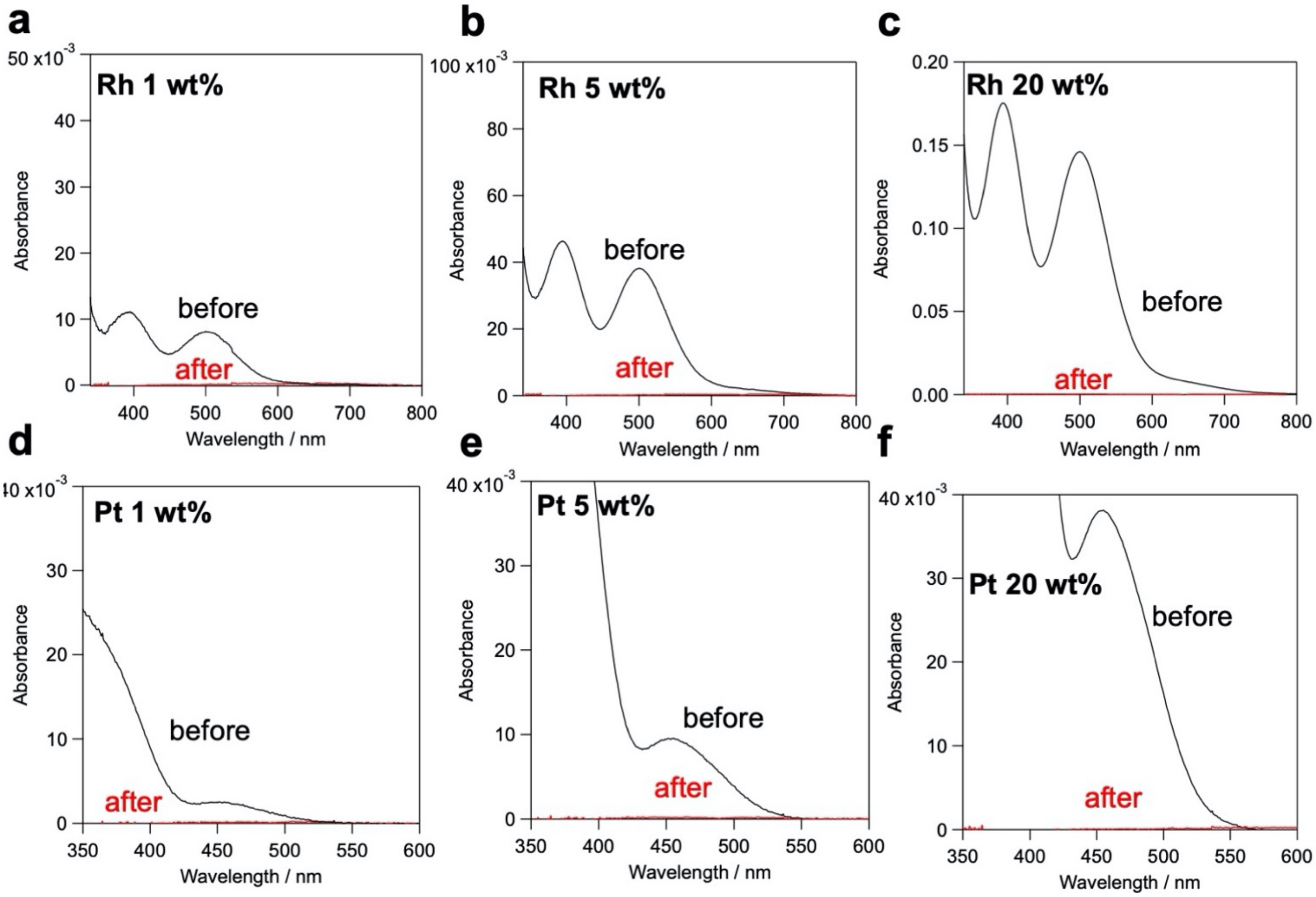

Fig. S8| Complete consumption of the metal precursors during PD process. Absorption spectra of precursors for cocatalysts ($RhCl_6^{3-}$ (**a-c**) and $PtCl_4^{2-}$ (**d-f**)) in the solutions before and after the PD process onto Bi_4NbO_8Cl (feed amount: 1–20 wt%).

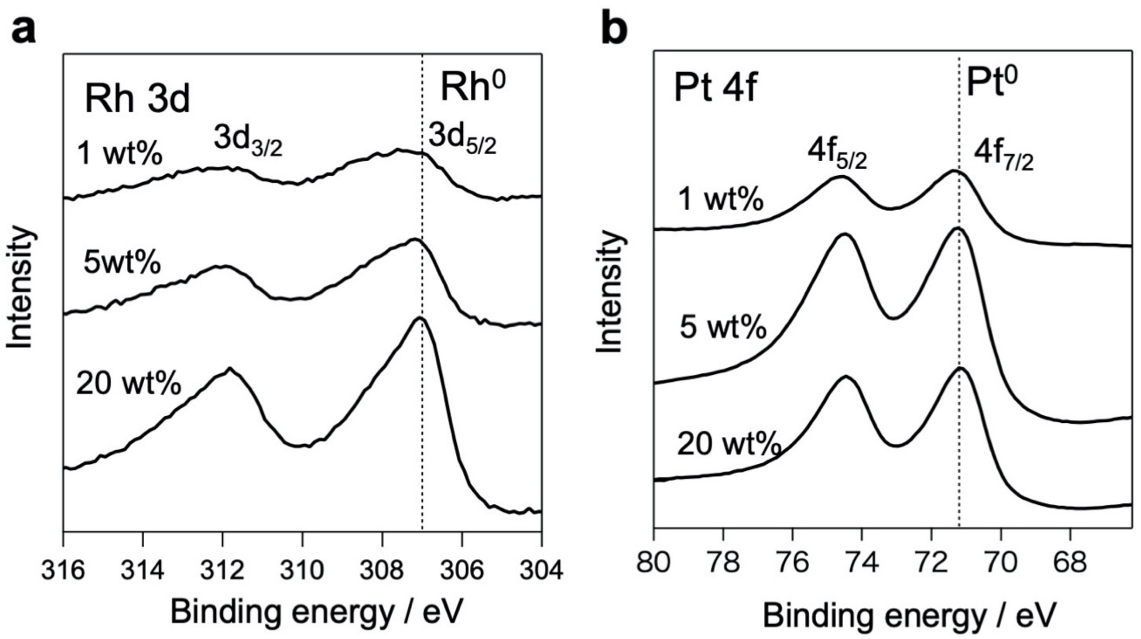

Fig. S9| Zero-valent state of the cocatalysts. XPS spectra of Rh-Bi_4NbO_8Cl and Pt-Bi_4NbO_8Cl in the Rh 3d (**a**) and Pt 4f (**b**) regions. The binding energies of elemental Rh and Pt were referred to from a reference handbook.[5]

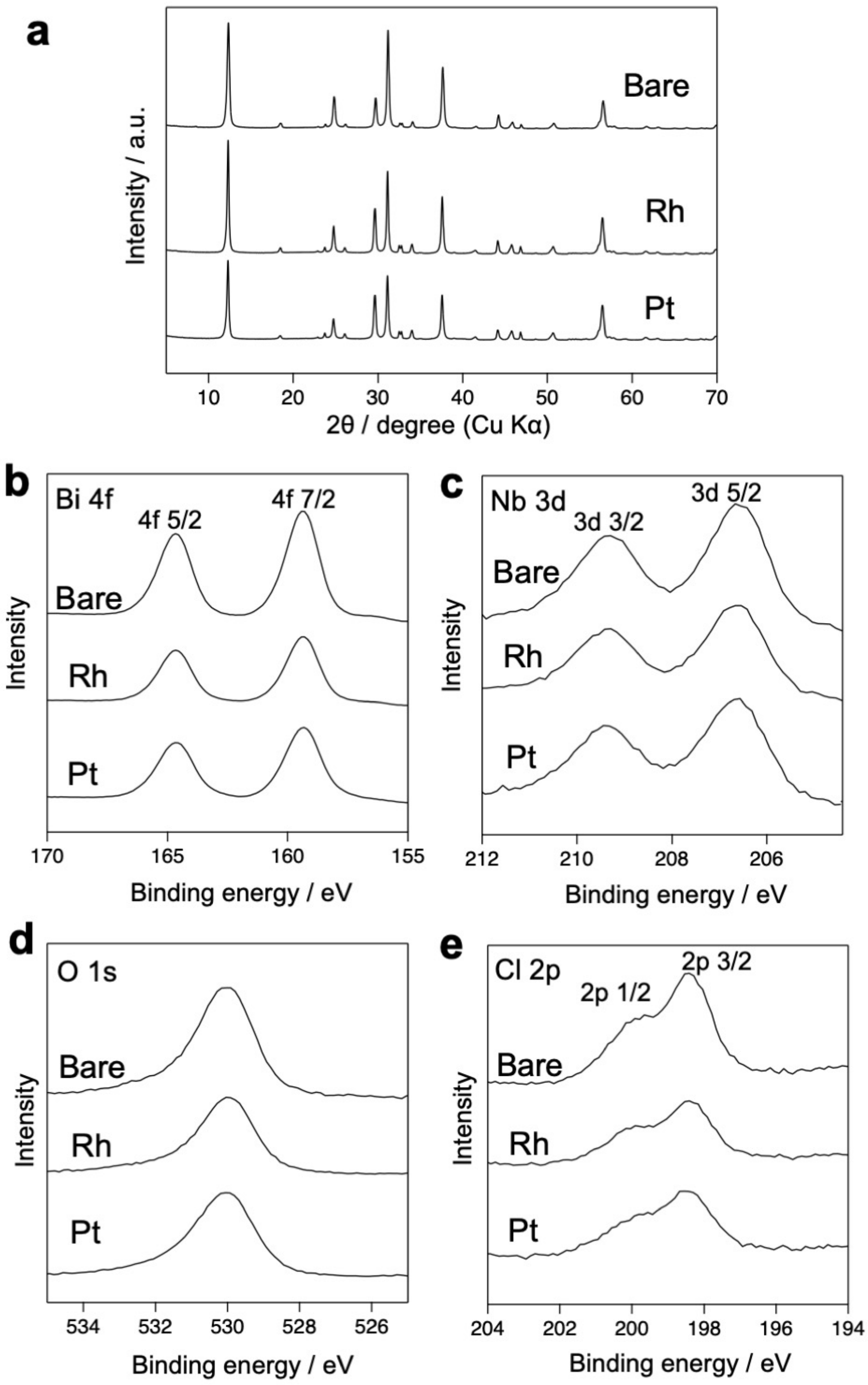

Fig. S10| Stability of the crystal phase and valence state after the cocatalyst loading. a, XRD patterns of Bare-, Rh-, and Pt-Bi_4NbO_8Cl. b-e, XPS spectra of Bare-, Rh-, and Pt-Bi_4NbO_8Cl in the Bi 4f (**b**), Nb 3d (**c**), O 1s (**d**), and Cl 2p (**e**) regions.

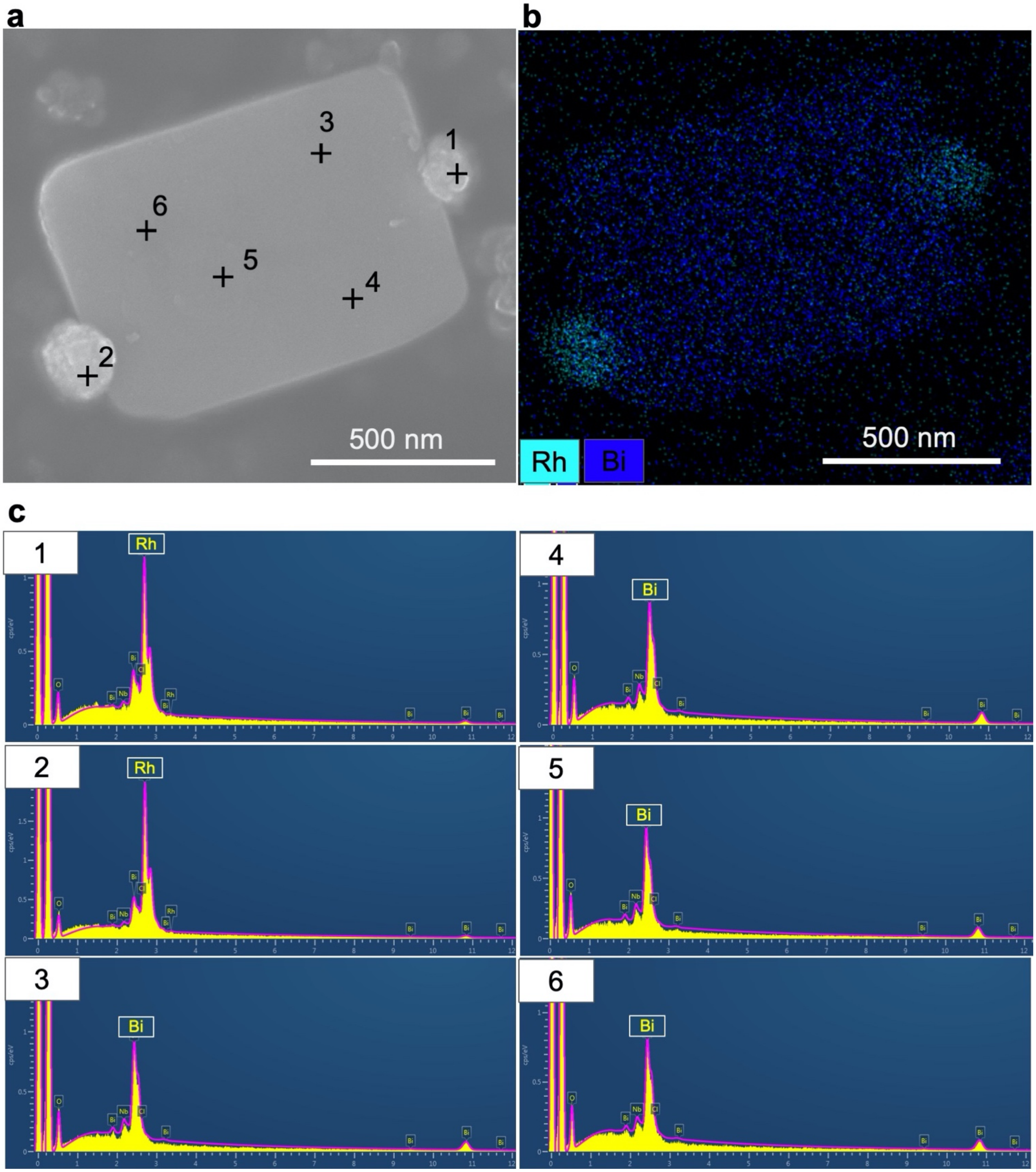

Fig. S11| Edge-selective deposition of the Rh. SEM image (**a**), SEM/EDX mapping (**b**), and SEM/EDX point analysis (**c**) for Rh-Bi_4NbO_8Cl.

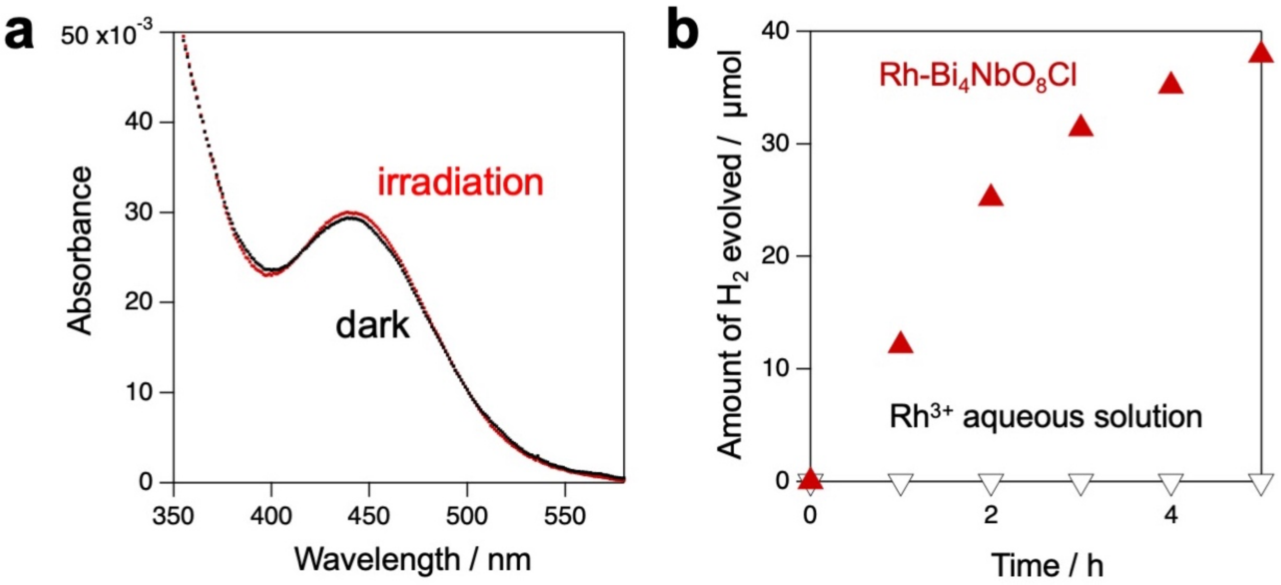

Fig. S12| Light irradiation without Bi_4NbO_8Cl photocatalyst. a, Absorption spectra of a solution containing the Rh^{3+} precursor (Na_3RhCl_6) after 5 h stirring with or without photoirradiation (λ > 400 nm). **b,** Time courses of H_2 evolution under light irradiation (λ > 400 nm) from a Na_3RhCl_6 methanol aqueous solution (hollow downward triangle) and **Rh-Bi_4NbO_8Cl** in a methanol aqueous solution (red upward triangles).

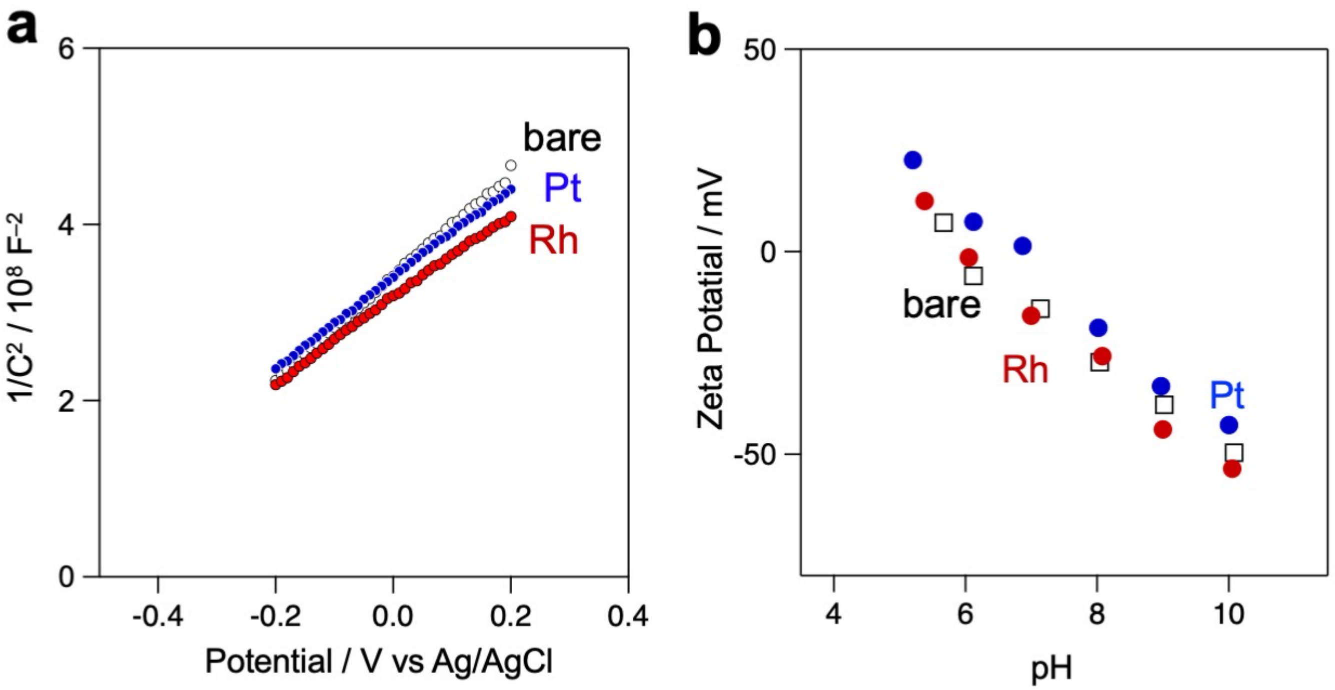

Fig. S13| Surface potential properties of Bare-, Pt- and Rh-Bi_4NbO_8Cl. a, Mott–Schottky plots in a 0.1 M phosphate buffer solution (pH = 6). **b**, Zeta potentials-pH plots for **Bare-Bi_4NbO_8Cl**, **Pt-Bi_4NbO_8Cl**, and **Rh-Bi_4NbO_8Cl**.

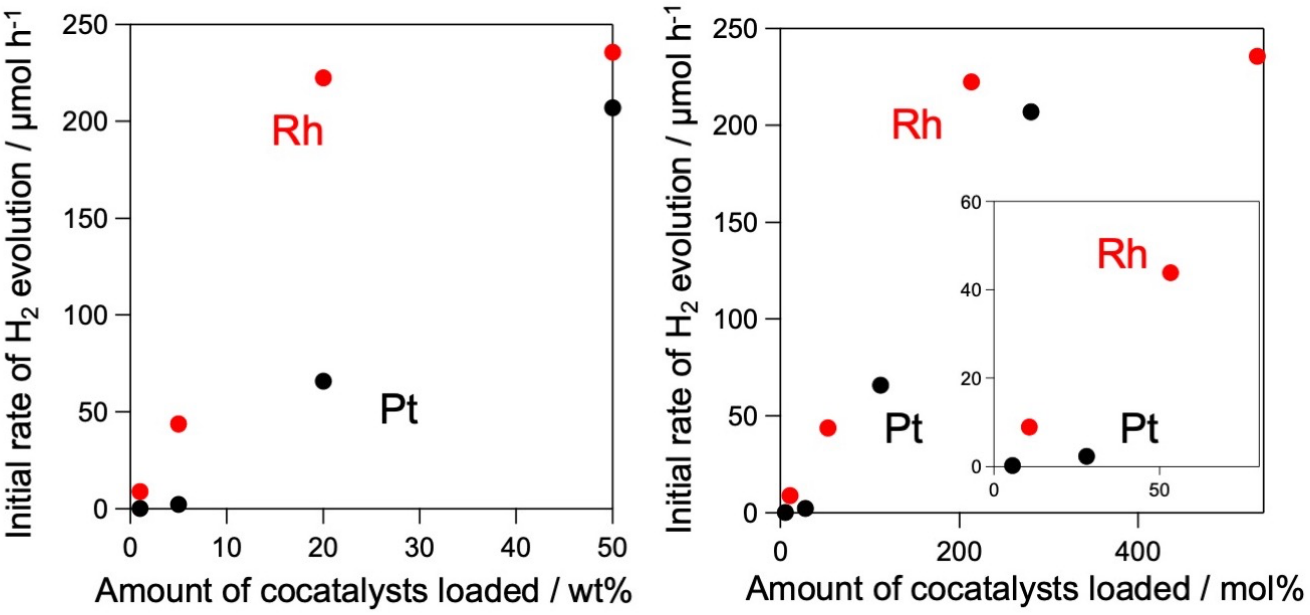

Fig. S14| Dependence of the loading amount of Rh or Pt cocatalysts on the H_2 evolution activity. Initial H_2 evolution rate over **Rh-Bi_4NbO_8Cl** and **Pt-Bi_4NbO_8Cl** is plotted versus the loading amount (in wt% (**a**), mol% (**b**)) of Rh and Pt. Light source, 300 W xenon lamp (λ > 300 nm); Medium, 100 mL of a methanol aqueous solution (20 vol%). In both plots, **Rh-Bi_4NbO_8Cl** clearly shows much higher H_2 evolution activity than **Pt-Bi_4NbO_8Cl** at a low loading amount, where Rh is selectively deposited on the edge while Pt is scattered on (001) of the Bi_4NbO_8Cl nanoparticle (Fig. S15). At high mol%, where Pt comes to be deposited on not only (001) surface but also edge, the H_2 evolution activity of **Pt-Bi_4NbO_8Cl** was not higher than **Rh-Bi_4NbO_8Cl,** despite better catalytic activity for HER of Pt than Rh.[6] This result indicates that Pt on (001), acting as a recombination site, affords a negative contribution to the H_2 evolution, while Pt of the edge provides a positive contribution to the H_2 evolution, showing the importance the deposition site of the cocatalyst for efficient H_2 evolution.

Supplementary Table 1 | AQE values for sacrificial hydrogen evolution reactions on semiconductor photocatalysts The AQE value of **Rh-Bi_4NbO_8Cl** (20 w% Rh) was greater than those in other photocatalytic systems excepting Rh-doped $SrTiO_3$, which is known to be an excellent photocatalyst.

Photocatalyst	BG/ eV		H_2 evolution	
			Activity / μmol h^{-1}	AQE / %
$SrTiO_3$:Rh[7]	2.3	MeOH	117	5.2 (420nm)
TaON[8]	2.5	MeOH	120	0.8 (420–500nm)
Ta_3N_5[9]	2.1	MeOH	10	0.1 (420-600 nm)
$LaTiO_2N$[10]	2.1	MeOH	30	0.15 (420 nm < λ < 600 nm)
$Sm_2Ti_2O_5S_2$[11]	2	MeOH	22	0.3 (440 nm < λ < 650 nm)
La–In oxysulfide[12]	2.6	MeOH	10	0.2 (420 nm < λ < 600 nm)
Bi_4NbO_8Cl (This work)	2.4	MeOH	93	2.6 (405 nm)

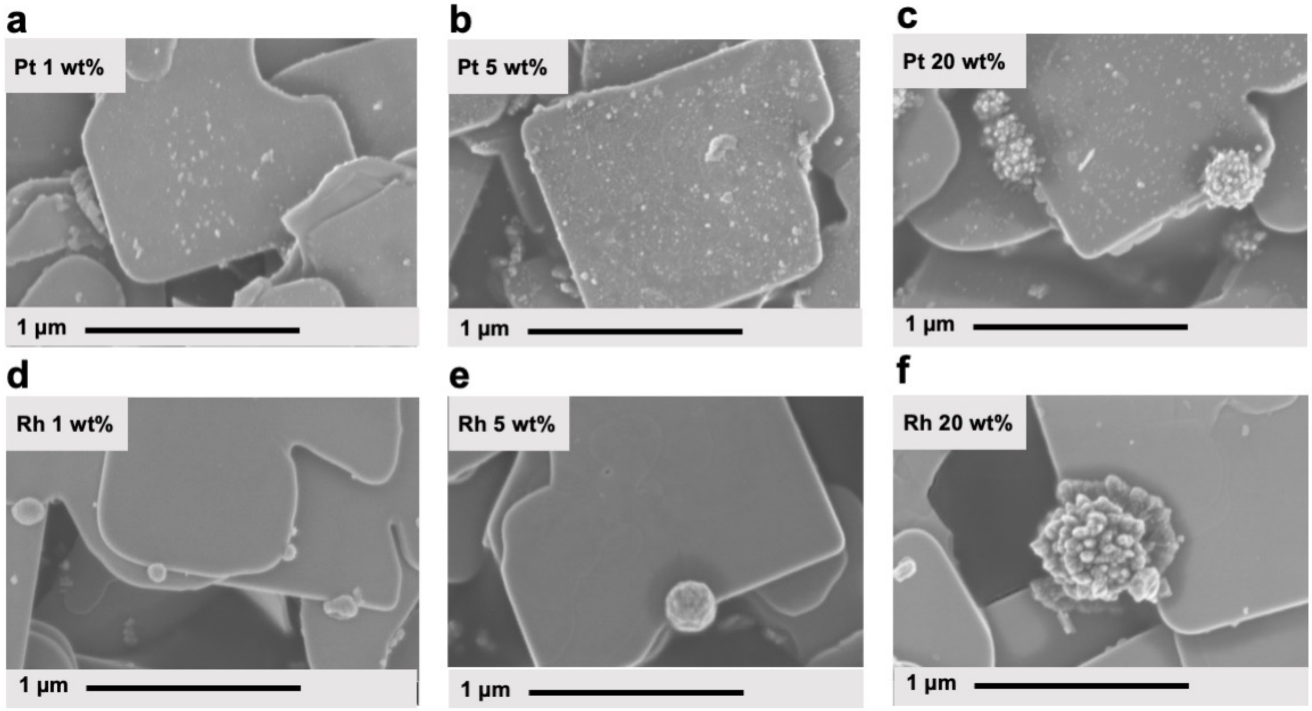

Fig. S15| Deposition site of Pt and Rh with various loading amount. SEM images Pt-Bi_4NbO_8Cl (**a-c**) and Rh-Bi_4NbO_8Cl (**d-f**) with various loading proportions (1–20 wt%).

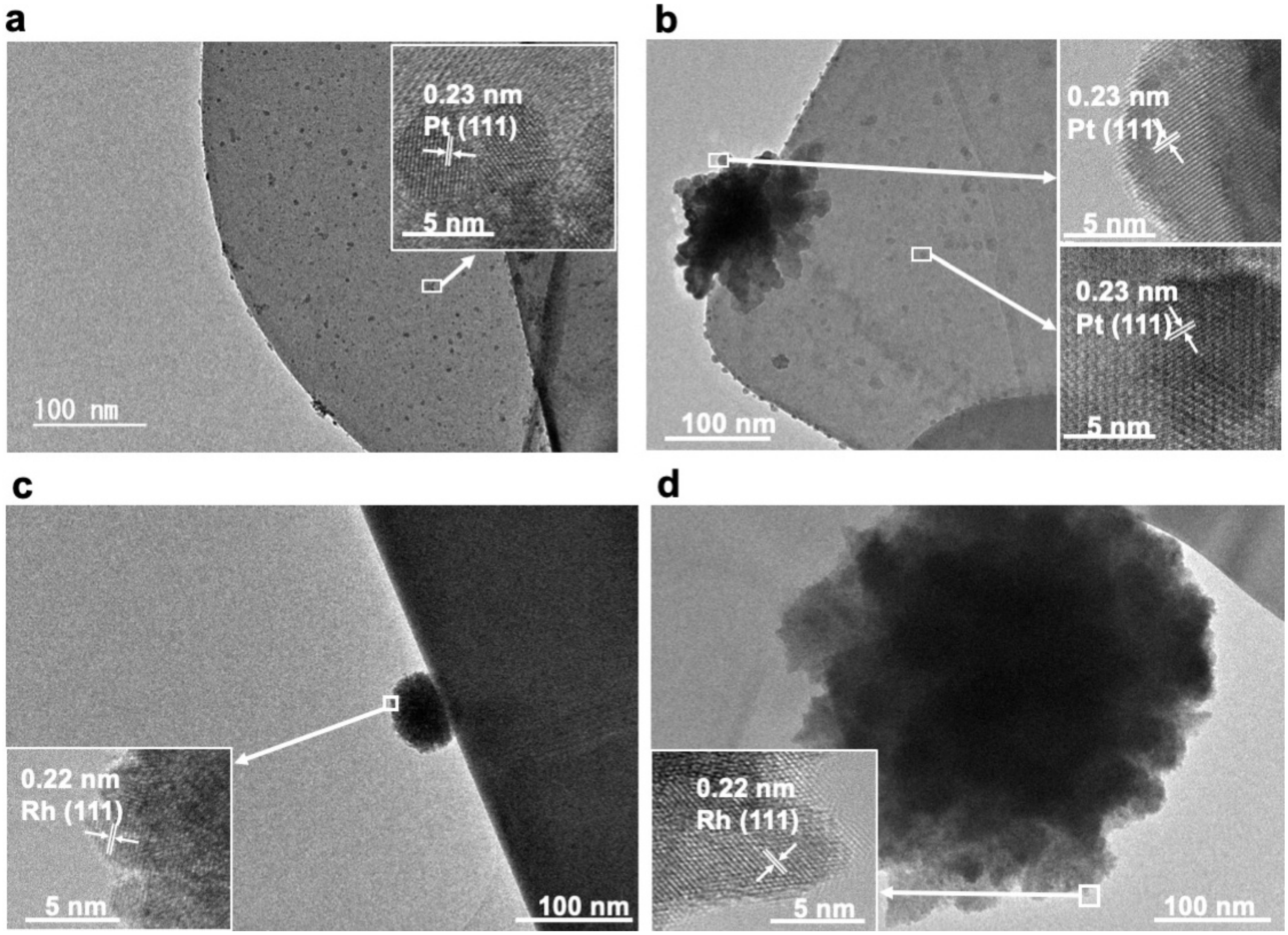

Fig. S16| Deposition site of Pt and Rh. a, **b**, HRTEM images of **Pt-Bi_4NbO_8Cl** with 1 wt% (**a**) and 20 wt% (**b**) of Pt loading. **c**, **d**, HRTEM images of **Rh-Bi_4NbO_8Cl** with 1 wt% (**c**) and 20 wt% (**d**) of Rh loading.

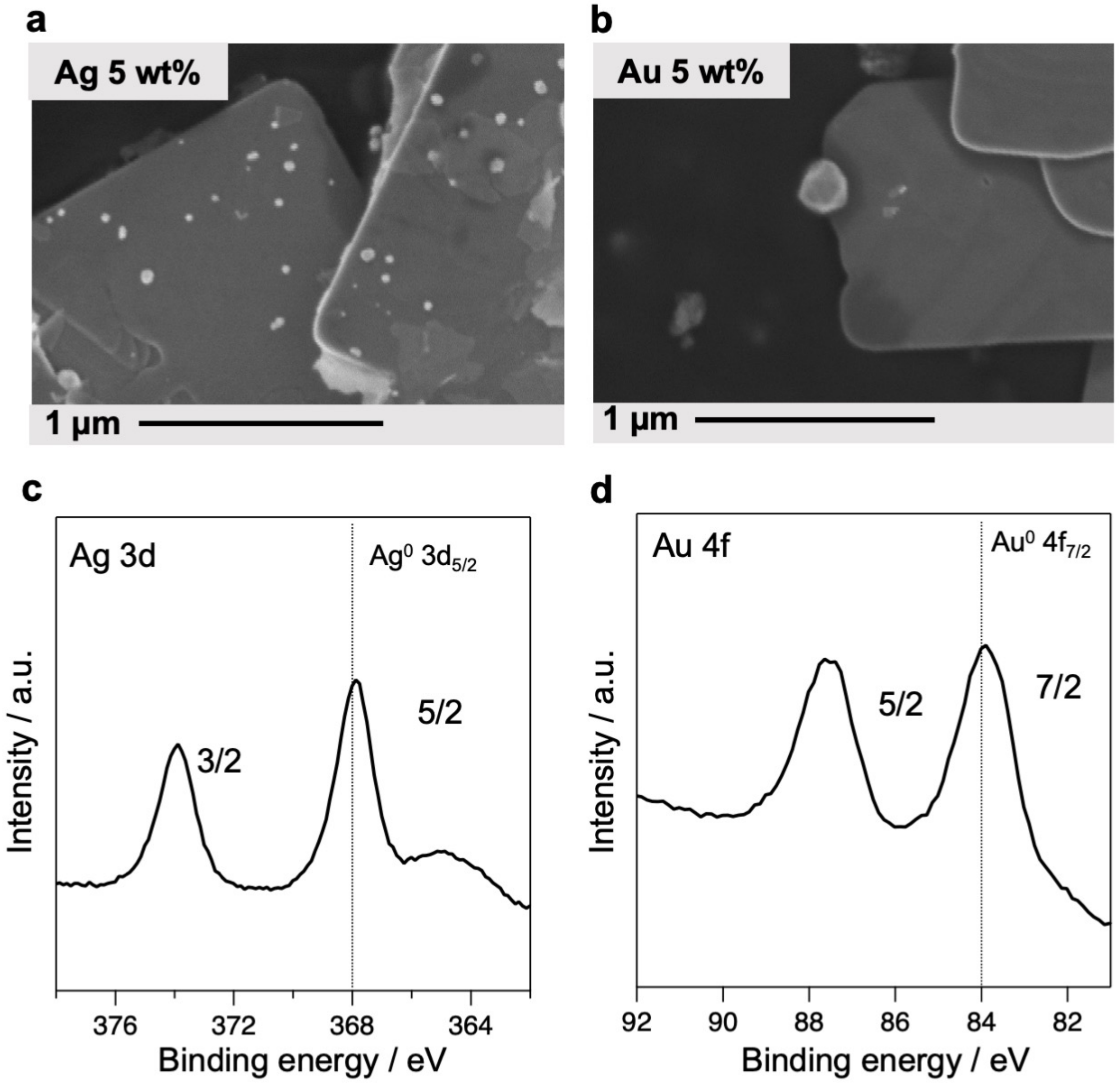

Fig. S17| Deposition-site selectivity depending on metal species. a, **b,** SEM images of Bi_4NbO_8Cl nanoplates loaded with Ag- (**a**) or Au- (**b**) via PD. 5 wt% of Ag and Au were loaded with $AgNO_3$ and $HAuCl_4$ as precursors, respectively. **c**, **d,** XPS spectra for Ag- and Au-loaded Bi_4NbO_8Cl nanoplates focusing on the Ag 3d (**c**) and Au 4f (**d**) region. The binding energies of Ag^0 and Au^0 were referred to from a reference hand book.[5] These results indicate a selectivity of deposition site depending on metal species. Although Au was deposited at the edge as Rh, the Au-loaded Bi_4NbO_8Cl showed a negligible activity (less than 0.01 μmol h^{-1}, Fig. S1), which is accounted for by the fact that the HER catalytic activity of Au is much inferior to that of Rh.[6] In other ward, Rh shows an appropriate deposition site as compared to Pt and possesses a much superior HER catalytic activity to Au, significantly enhancing the photocatalytic activity of Bi_4NbO_8Cl.

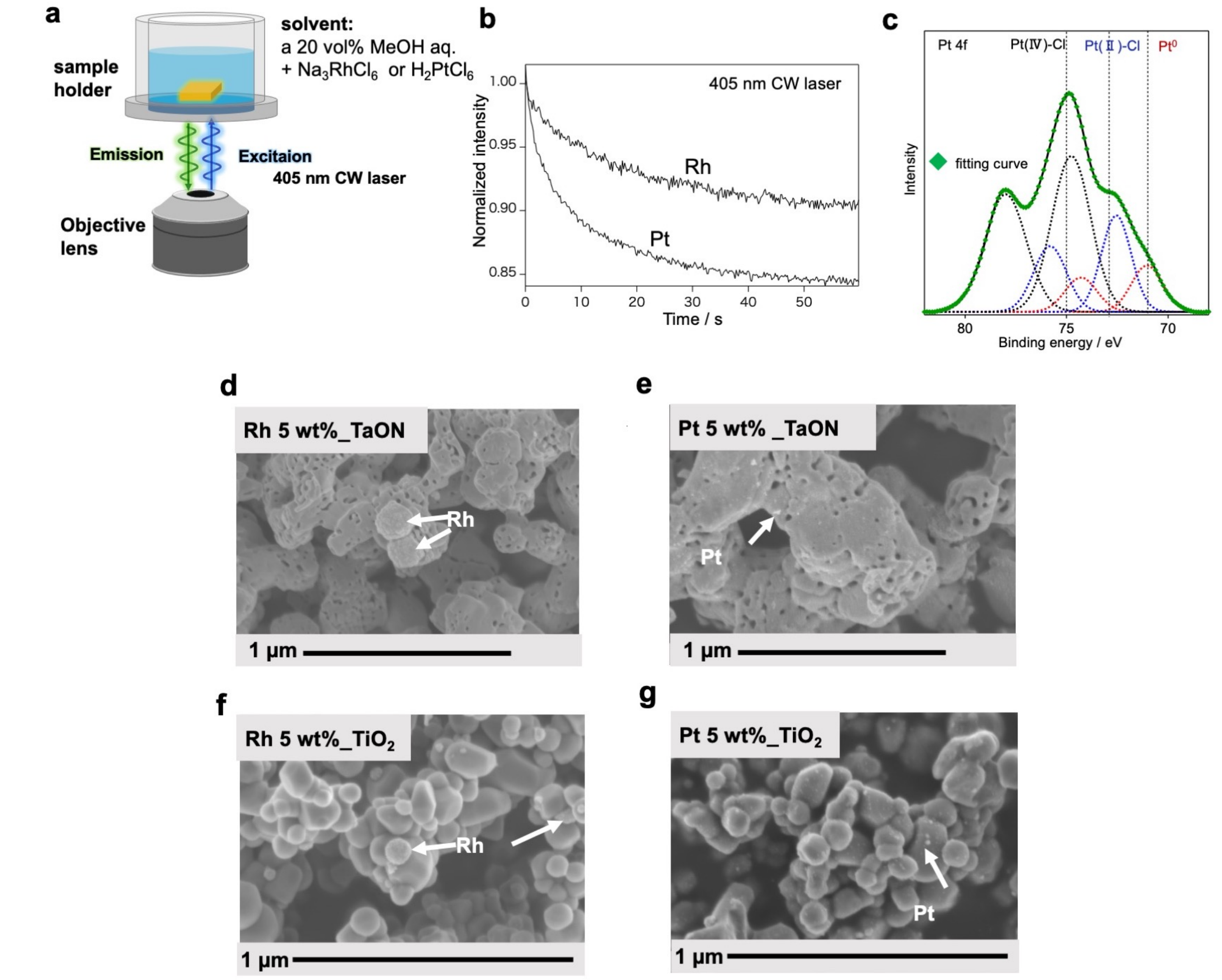

Fig. S18| Plausible factors for metal-dependent site-selective deposition. a, Experimental setup to acquire time-course PL decays by the metal deposition on Bi_4NbO_8Cl. **b**, Decay in the PL intensity of Bi_4NbO_8Cl in the presence of Pt^{4+} or Rh^{3+} under 405 nm laser irradiation. **c**, XP spectrum focusing on the Pt 4f region for Bi_4NbO_8Cl after 5 min in the Pt photo-deposition process (λ > 400 nm, 300 W Xe-lamp). The binding energies of the Pt species were referred to a previous literature.[13] **e-g**, SEM images Rh- and Pt-TaON (**d**,**e**) and Rh- and Pt-TiO_2 (**f**,**g**).

The PL intensity decay in **b** was much faster in the presence of Pt^{4+}than Rh^{3+}. This indicates that Pt^{4+} is reduced faster than Rh^{3+} by photoexcited electrons in Bi_4NbO_8Cl. In general, a greater reduction rate of metal produces smaller particles on photocatalyst.[14,15] Therefore, we conclude that the reduction rate of the Pt^{4+} or Rh^{3+} is a key determining their deposition sites. In other words, while Pt^{4+} can be easily reduced and deposited without site selectiveness, Rh^{3+} is reduced at a slower rate to be selectively

deposited on the edge where electrons accumulate. The Rh deposited via the impregnation method are not selectively on the edge of the particle, indicating that the specific absorption property of Rh may not determine the deposition site as shown in Fig. S19. In addition, we have conducted Pt and Rh photo-deposition on TaON and TiO_2, both of which are often-used photocatalysts. As shown in **d**–**g**, Rh was deposited aggregately, while Pt were highly dispersive on TaON and TiO_2. These results indicate that the reactivity of the Pt^{4+} and Rh^{3+} itself affects the photoreduction process and reduction sites.

Although further investigation is required to draw a concrete conclusion, the number of electrons participating in the reduction of metal ions might be associated with the reduction rates. The numbers of electrons to be required for the each reduction step of a metal ion, are greater for Rh and Au than for Pt and Ag: $[Rh^{III}Cl_6]^{3-} + 3e^- \rightarrow Rh + 6Cl^-$; $[Au^{III}Cl_4]^- + 3e^- \rightarrow Au + 4Cl^-$; $[Pt^{IV}Cl_6]^{2-} + 2e^- \rightarrow [Pt^{II}Cl_4]^{2-} + 2Cl$ and $[Pt^{II}Cl_4]^{2-} + 2e^- \rightarrow Pt + 4Cl^-$; $Ag^+ + e^- \rightarrow Ag$. Note that Pt^{II} was the genuine intermediate for the photodeposition process of Pt^{IV}, which was clarified by the XP spectrum measured for a Bi_4NbO_8Cl sample in the early stage of the photodeposition (**c**).

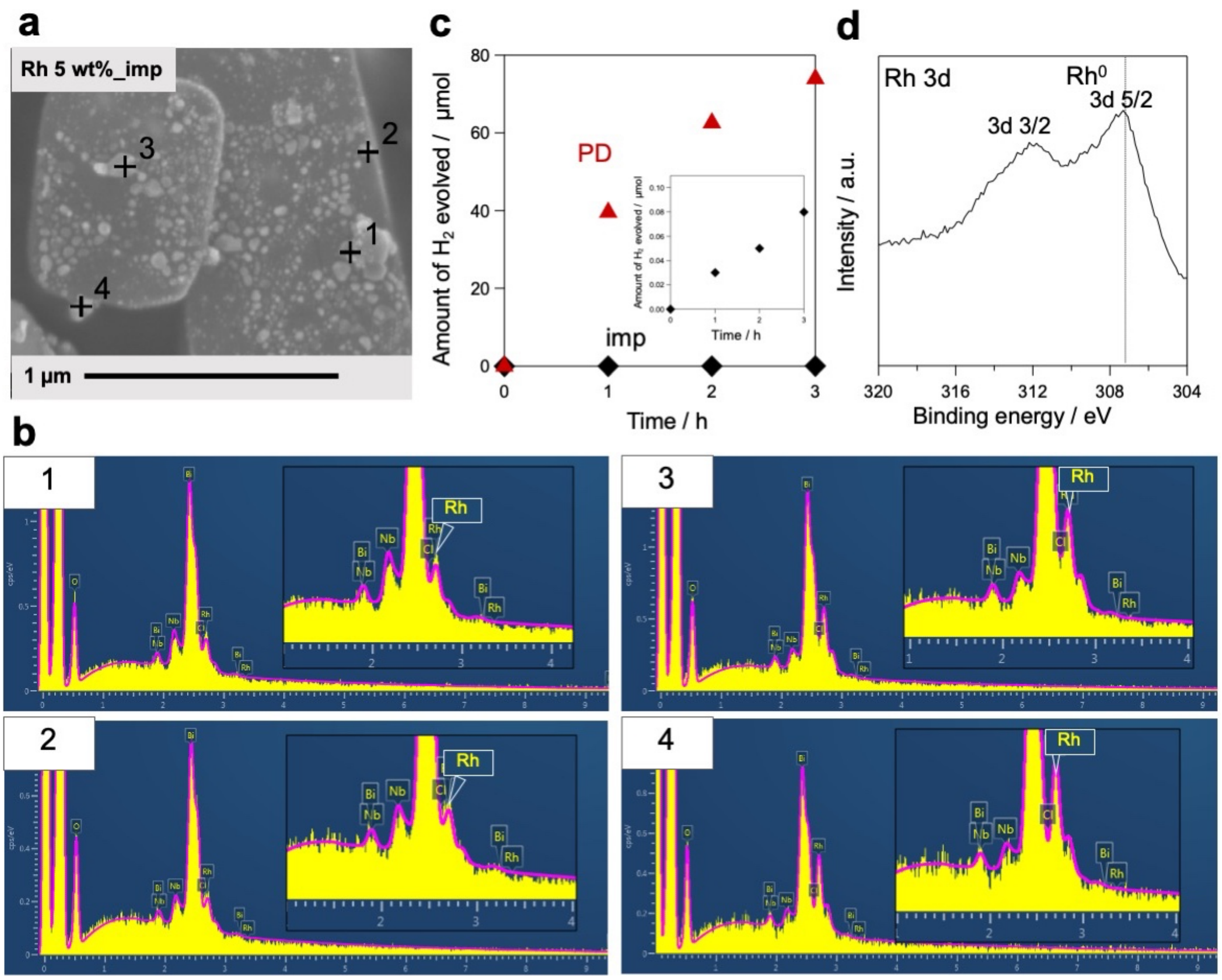

Fig. S19| Rh deposition via the impregnation method. SEM images (**a**) and SEM/EDX point analysis (**b**) of a Rh-loaded Bi_4NbO_8Cl sample (5 wt%) by the impregnation method. **c,** Time courses of H_2 evolution in a methanol aqueous solution (20 vol%) under visible light irradiation (λ > 400 nm). **d,** XPS spectrum for a Rh-loaded Bi_4NbO_8Cl sample by the impregnation method in the Rh 3d region. Note that the impregnation method often affords random depositions, because this process is initiated by the "forced" adsorption of metal cations onto photocatalyst surfaces by solvent evaporation, followed by thermal reduction.

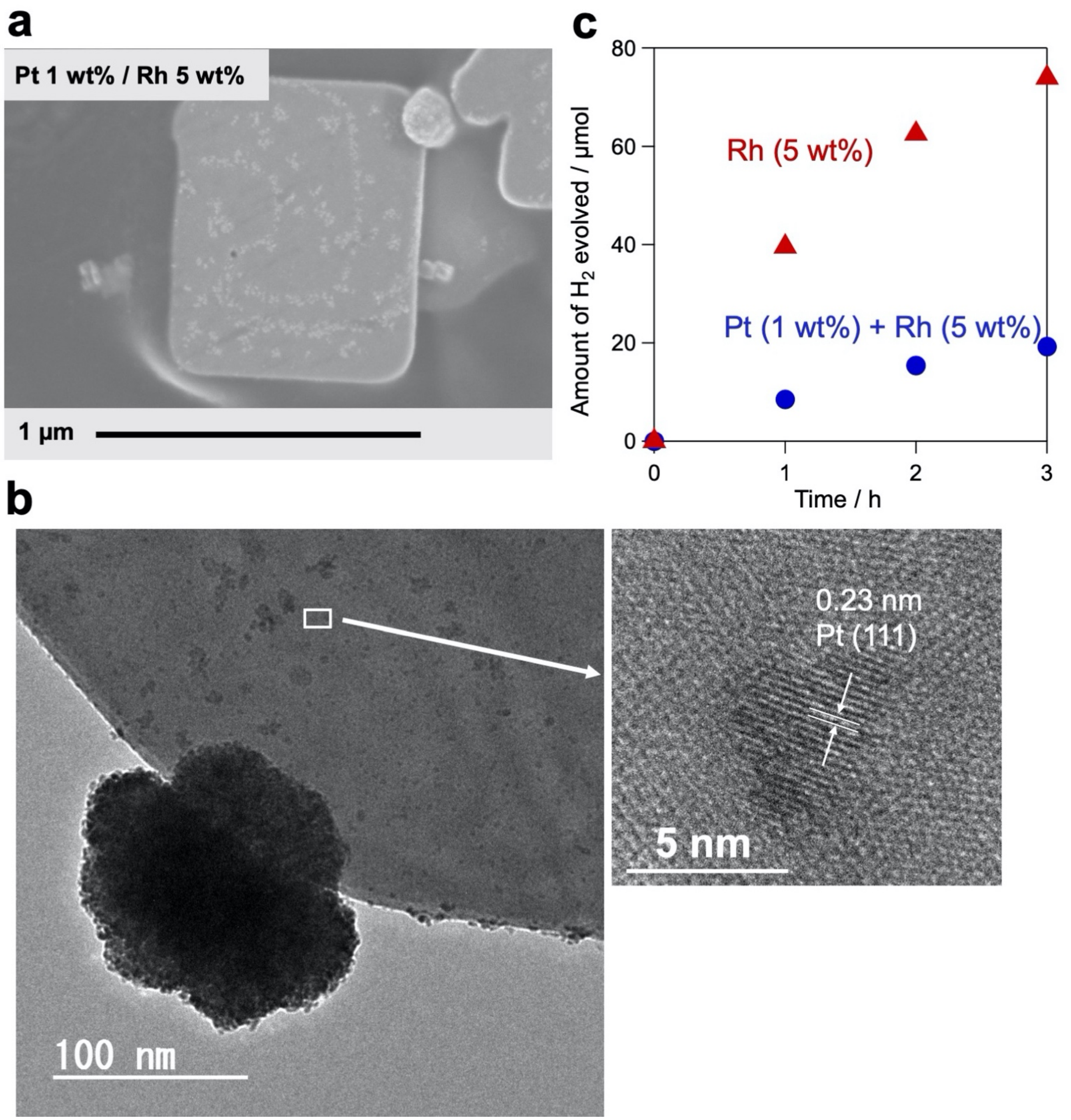

Fig. S20| Co-deposition of Rh and Pt by PD. SEM (**a**) and HRTEM (**b**) images of Pt (1 wt%)-loaded Rh-Bi_4NbO_8Cl (5 wt% Rh). **c,** Time courses of H_2 evolution over Pt (1 wt%)-loaded Rh-Bi_4NbO_8Cl (5 wt% Rh) in a methanol aqueous solution (20 vol%) under light irradiation ($\lambda > 300$ nm).

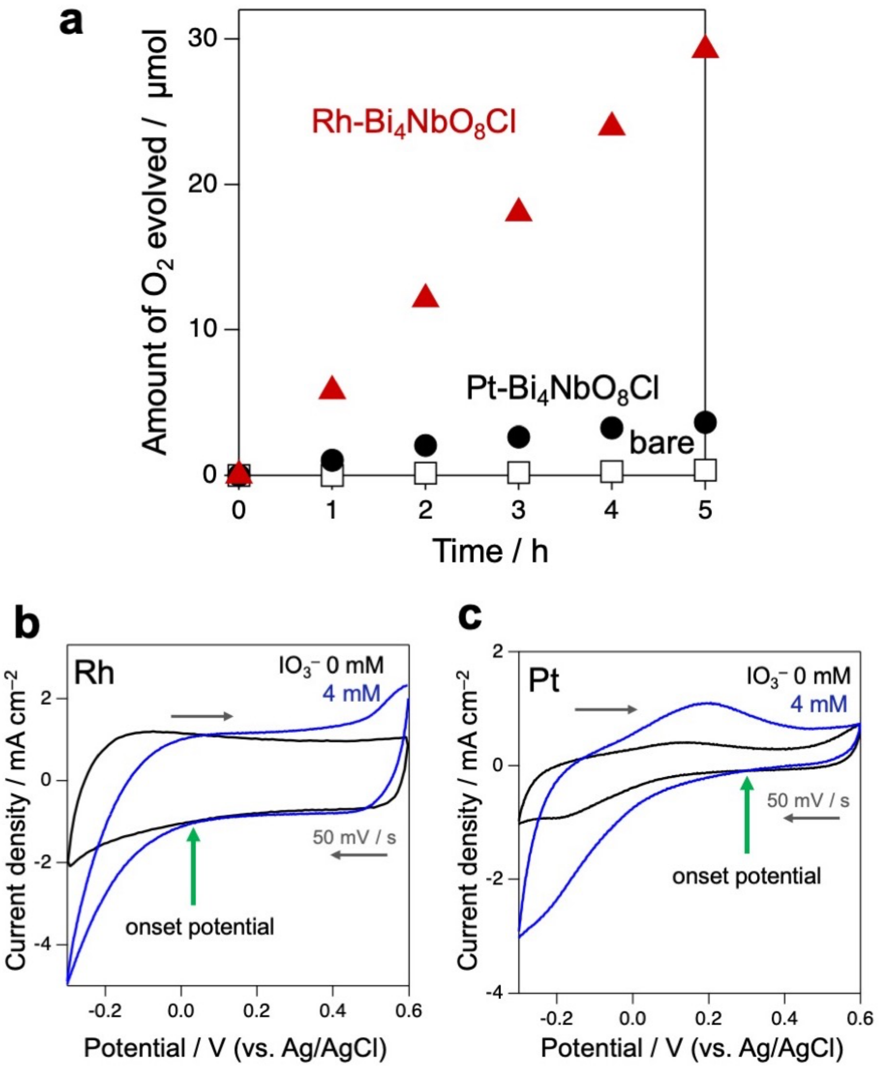

Fig. S21| Photocatalytic water oxidation activity. **a**, Time courses of O_2 evolution over Bare-Bi_4NbO_8Cl (squares), Rh-Bi_4NbO_8Cl (triangles) and Pt-Bi_4NbO_8Cl (circles) in in aqueous $NaIO_3$ solution (4 mM) under visible-light ($\lambda > 400$ nm) irradiation. **b**, **c**, Cyclic voltammogram of FTO substrates loaded with Rh (b) or Pt (c) in 0.5 M aqueous Na_2SO_4 solution with or without 4 mM IO_3^-, at a scan rate of 50 mVs^{-1}. The Pt and Rh electrodes ware prepared via the drop-casting method on a fluorine-doped tin oxide (FTO) glass electrode. A small amount of H_2PtCl_6 or Na_3RhCl_6 aqueous solution was spread on an FTO substrate. The substrate was dried in air at room temperature and subsequently calcined under an H_2 flow (20 mL min^{-1}) at 473 K for 30 min. Electrochemical measurements were performed using a potentiostat (VersaSTAT4, Princeton Applied Research Co., Ltd.) and a cell consisting of a prepared electrode, Pt wire, and Ag/AgCl electrode as the working electrode, counter electrode, and the reference electrode, respectively.

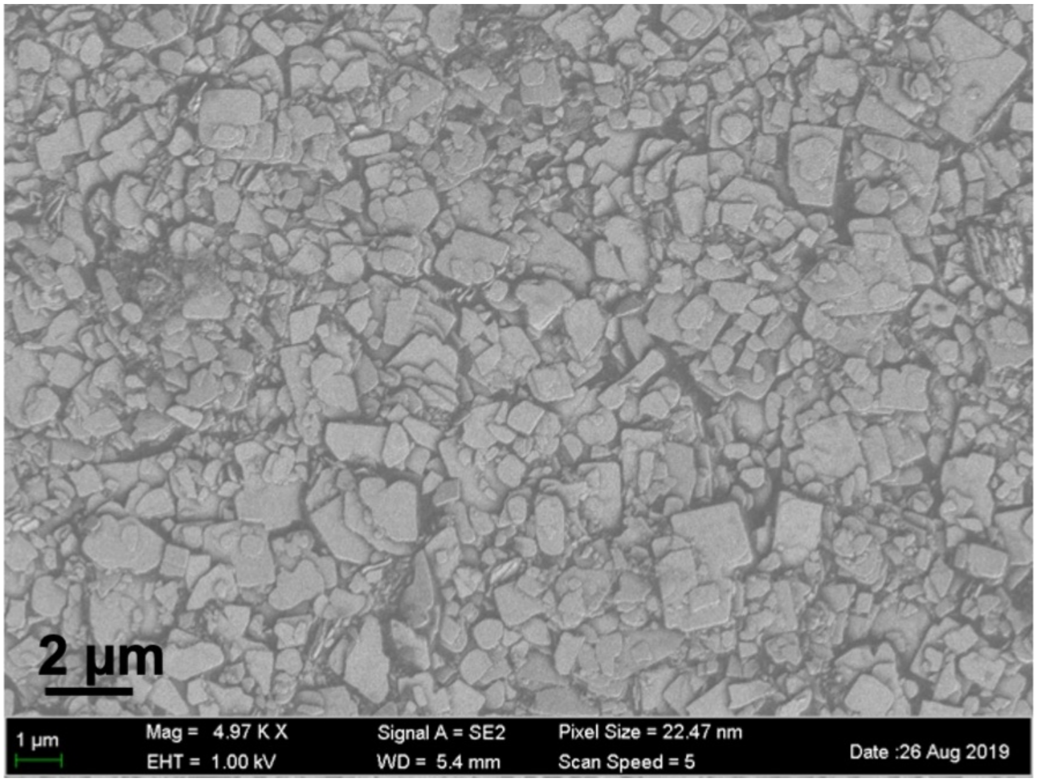

Fig. S22| Bi_4NbO_8Cl nanoplates fixed in the face-to-face orientation against the substrate. SEM image of the Bi_4NbO_8Cl sample deposited on a quartz substrate. Simple deposition of Bi_4NbO_8Cl sheets onto a quartz substrate resulted in a fixed, face-to-face orientation against the substrate, which allowed us to evaluate the anisotropy of carrier mobility in directions perpendicular and parallel to the (001) plane.

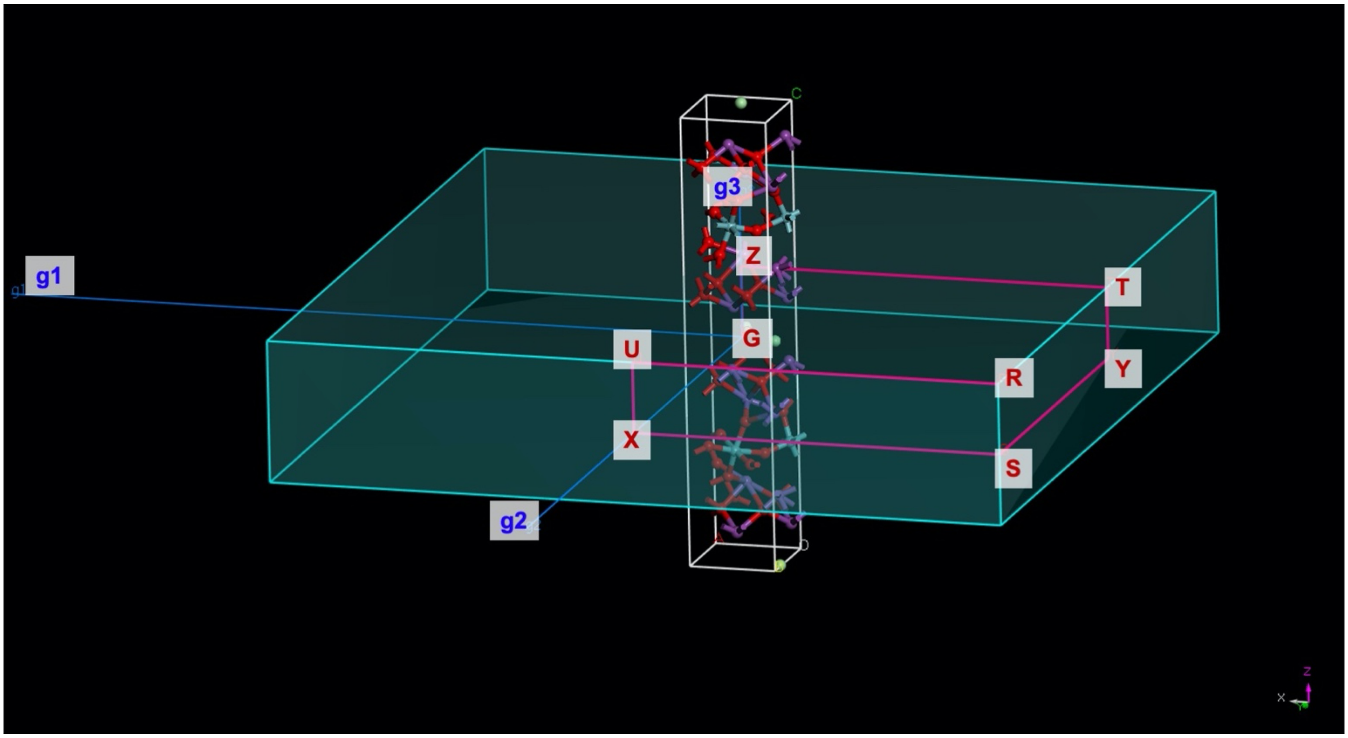

Fig. S23| First Brillouin zone of Bi_4NbO_8Cl. The magenta line corresponds to the path of the band structure diagram, and g1, g2, and g3 denote the reciprocal vectors.

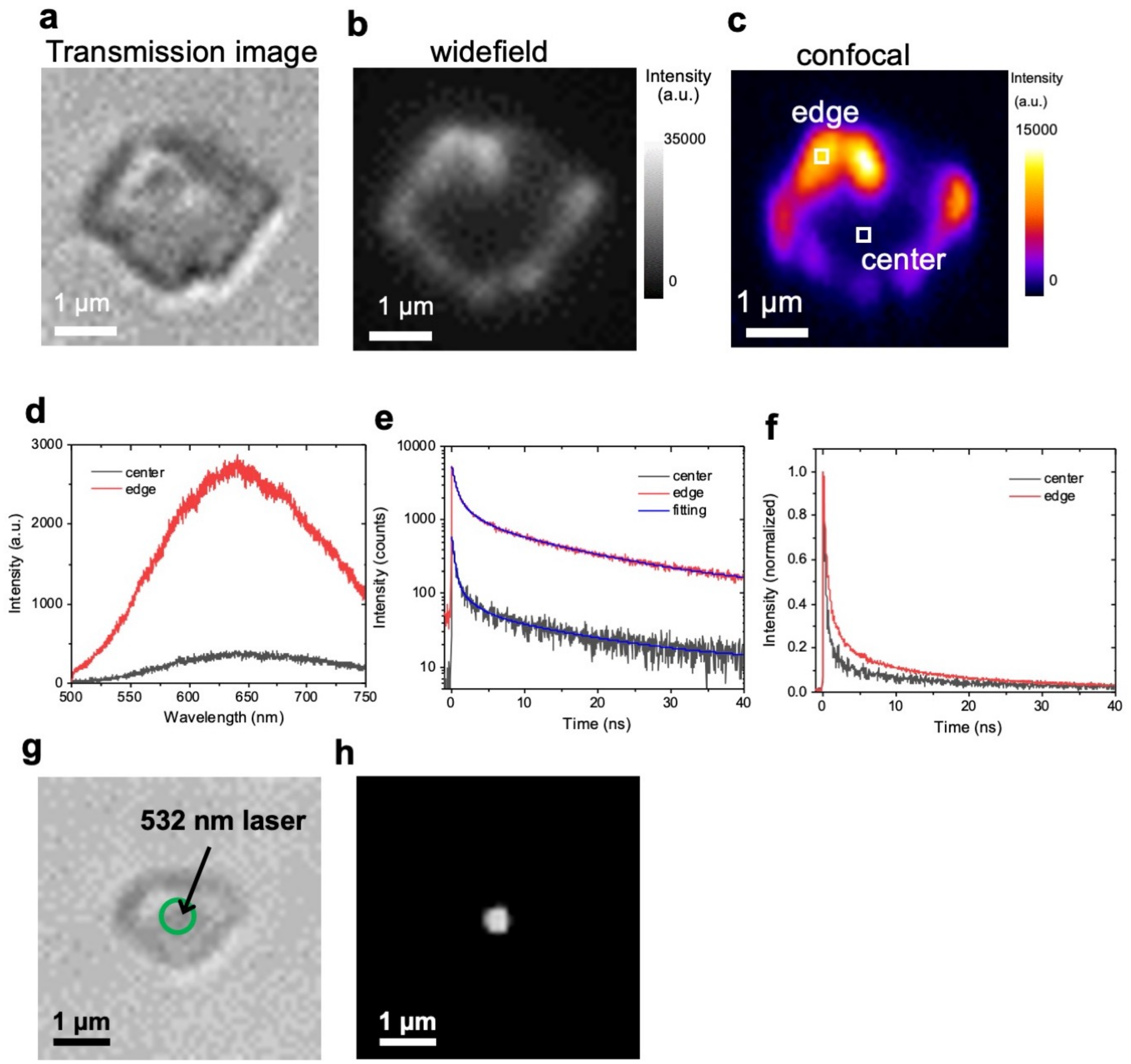

Fig. S24| Single-particle PL imaging. Transmission (**a**) and PL images (**b, c**) of a single **Bare-Bi_4NbO_8Cl** nanoplate immobilized on a cover glass in air under 405 nm laser irradiation. The PL intensity was integrated over all monitored wavelengths (> 500 nm). **d**-**f**, PL spectra (**d**) and decays (**e**, **f**) from the edge and center of the nanoplate monitored in the white boxes in **c**. **g**, A transmission image of a single **Bare-Bi_4NbO_8Cl** nanoplate immobilized on a cover glass in air. The illuminated region by a 532 nm laser in **h** is shown by the green circle. **h,** Another image of the same region taken in the dark except for the illumination by a focused 532 nm laser. The full width at half maximum (FWHM) of the bright spot on the crystal is approximately 570 nm, which was determined by Gaussian fitting.

The PL image shown in Fig. 4a is a wide-field image in which the entire particle was photoexcited at once. On the other hand, here we employed confocal microscopy technique, where the luminescence was recorded point-by-point (100.7 nm intervals) under localized laser excitation. The PL intensity was much greater at the edge than at the basal plane. Moreover, decay profiles and lifetimes are different between luminescence at the edge and that from the center of the (001) plane. In addition, when only the center of a Bi_4NbO_8Cl nanoplate was irradiated by a focused 532 nm CW laser, at which wavelength Bi_4NbO_8Cl has a negligible absorption (Fig. S3), the incident light did not propagate to the edges of the nanoplate (**h**). From these experimental facts, we have concluded the PL observed at the edge originates from the edge itself, and there is a minor contribution from luminescence transmitted from the bulk part of Bi_4NbO_8Cl through the optical waveguide mode.

Supplementary Table 2| Fitting parameters for the PL decays.

	a_1	τ_1 (ns)	a_2	τ_2 (ns)	a_3	τ_3 (ns)	**<τ> (ns)**[a]
center	1697	0.38	109	2.0	53	15.4	7.9
edge	4971	0.52	1354	2.3	923	14.6	10.7

[a] $\langle\tau\rangle = (a_1\tau_1^2 + a_2\tau_2^2 + a_3\tau_3^2)/(a_1\tau_1 + a_2\tau_2 + a_3\tau_3)$

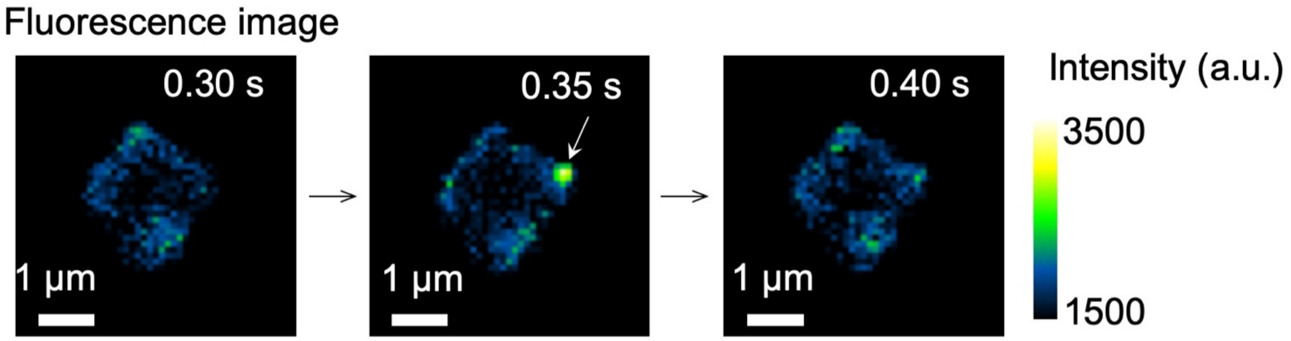

Fig. S25| Fluorescence burst observed during the single-particle fluorescence imaging experiment. The green fluorescence from individual product molecules appeared as burst-like signals due to dissociation from the surface into the bulk solution.[16]

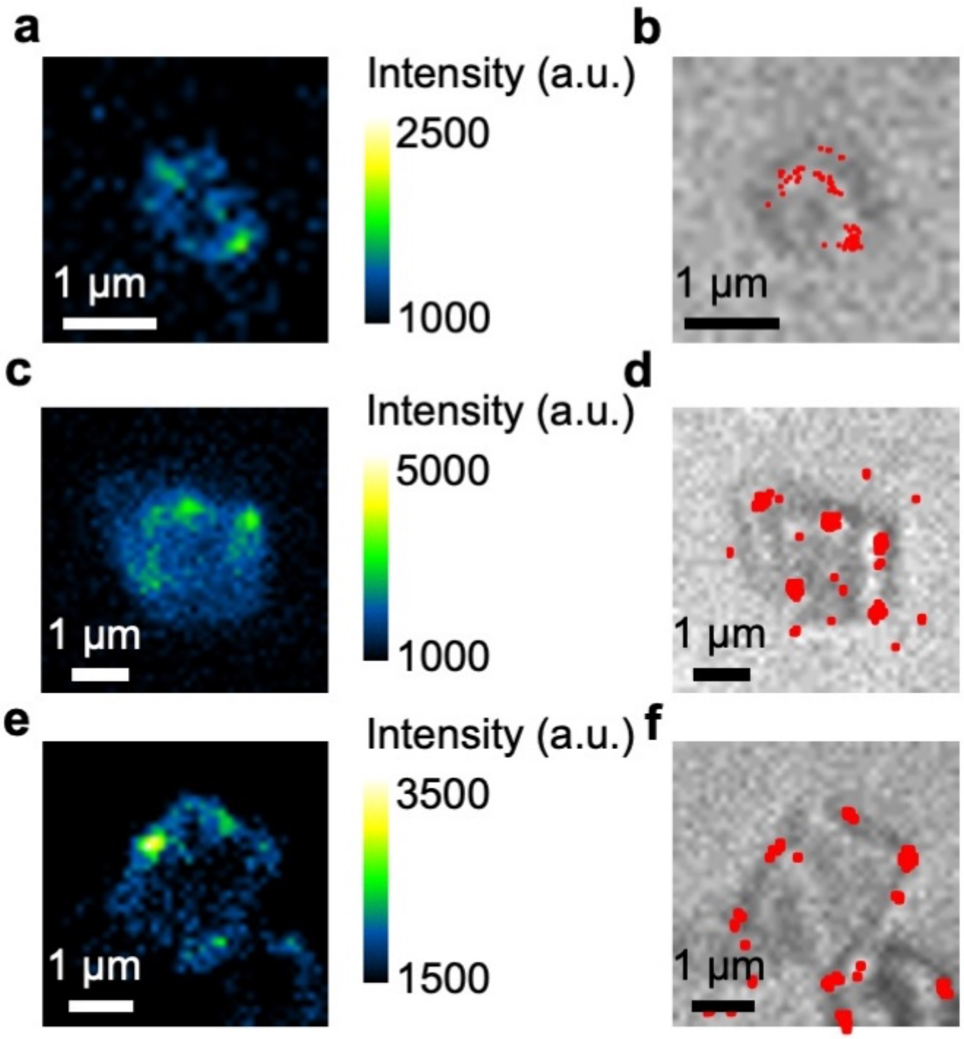

Fig. S26| Single-particle fluorescence images for other Bi_4NbO_8Cl nanoplates. a, c, e, The fluorescence images were taken under 488 nm laser irradiation. **b, d, f,** The red dots represent fluorescence bursts observed during 180 s irradiation.

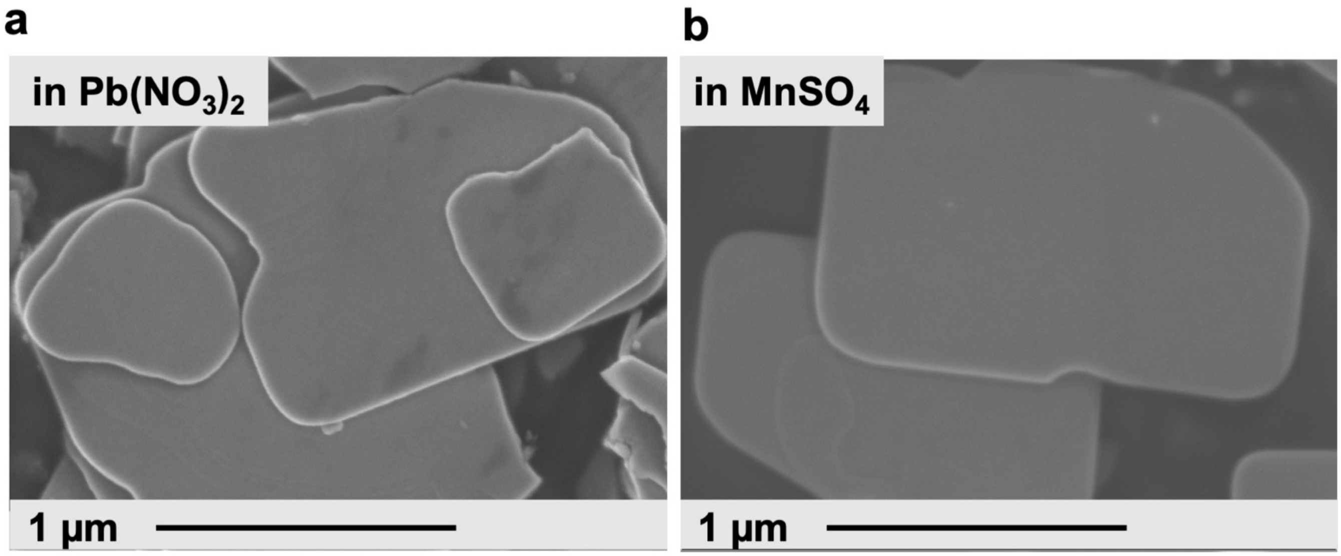

Fig. S27| Absence of PbO_2 or MnO_4 on Bare-Bi_4NbO_8Cl after PD. SEM images of the **Bare-Bi_4NbO_8Cl** after visible light irradiation in $Pb(NO_3)_2$ (**a**) and $MnSO_4$ (**b**) solutions with O_2 bubbling.

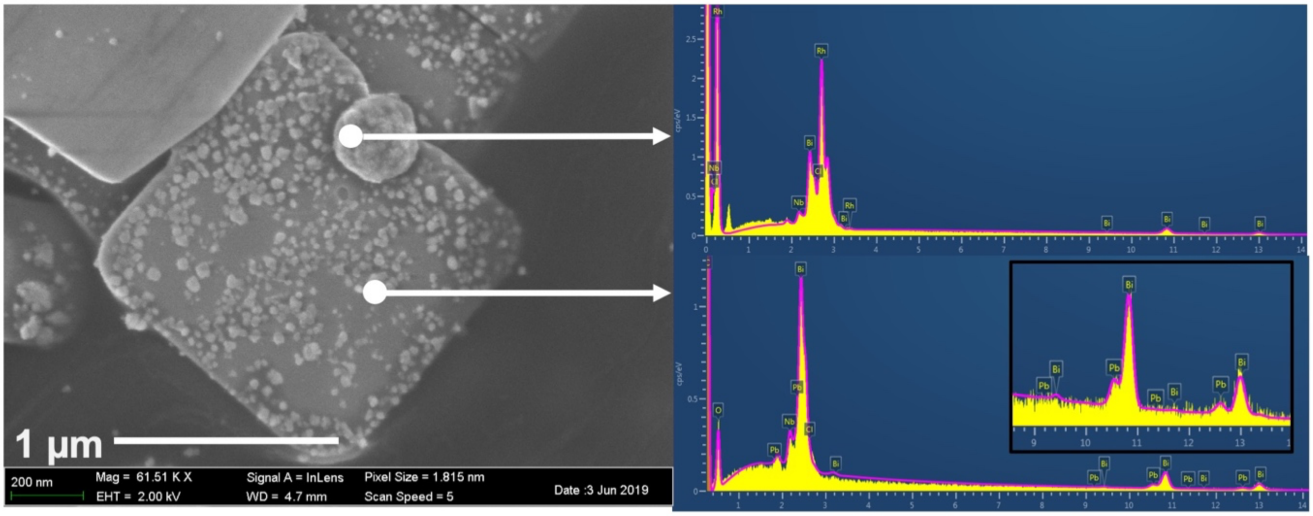

Fig. S28| SEM image and SEM/EDX analysis for Rh-Bi_4NbO_8Cl after the photocatalytic deposition of PbO_2.

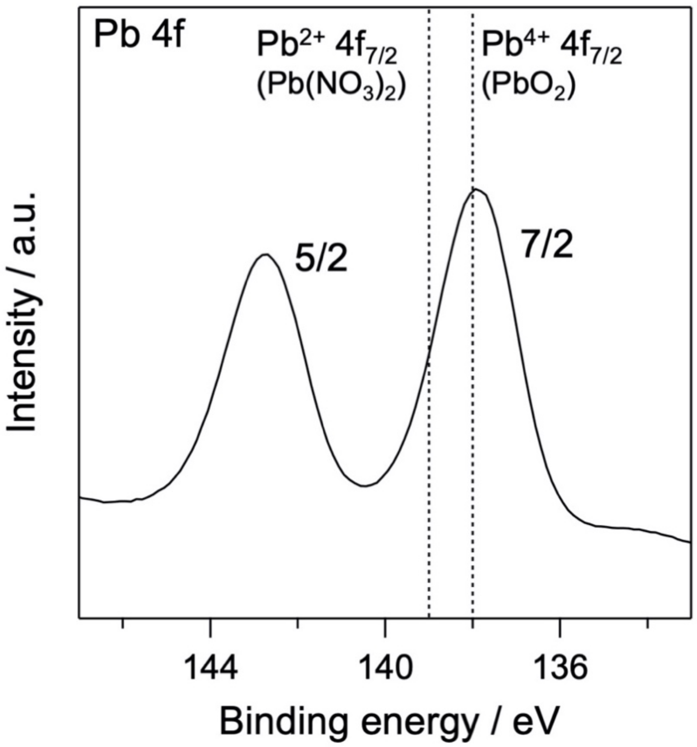

Fig. S29| Valence state of photo-deposited Pb species. XPS spectra of **Rh-Bi_4NbO_8Cl** after the photo-catalytic deposition of PbO_2 in the Pb 4f region. The binding energies of Pb^{2+} and Pb^{4+} were referred to from a reference hand book.[5]

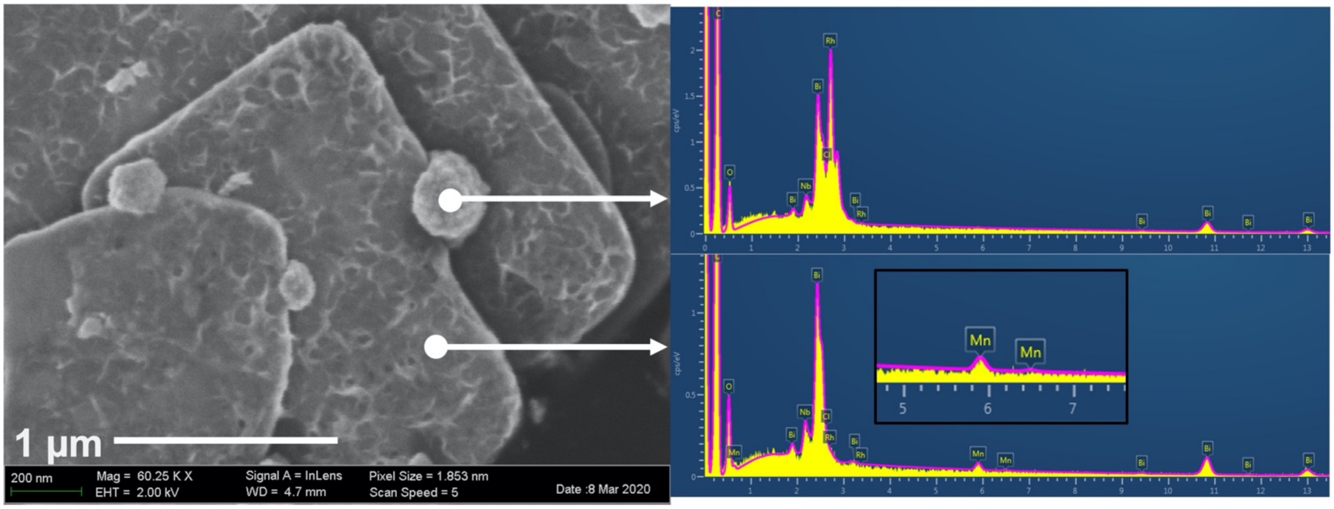

Fig. S30| SEM image and SEM/EDX analysis for Rh-Bi_4NbO_8Cl after the photocatalytic deposition of MnO_2.

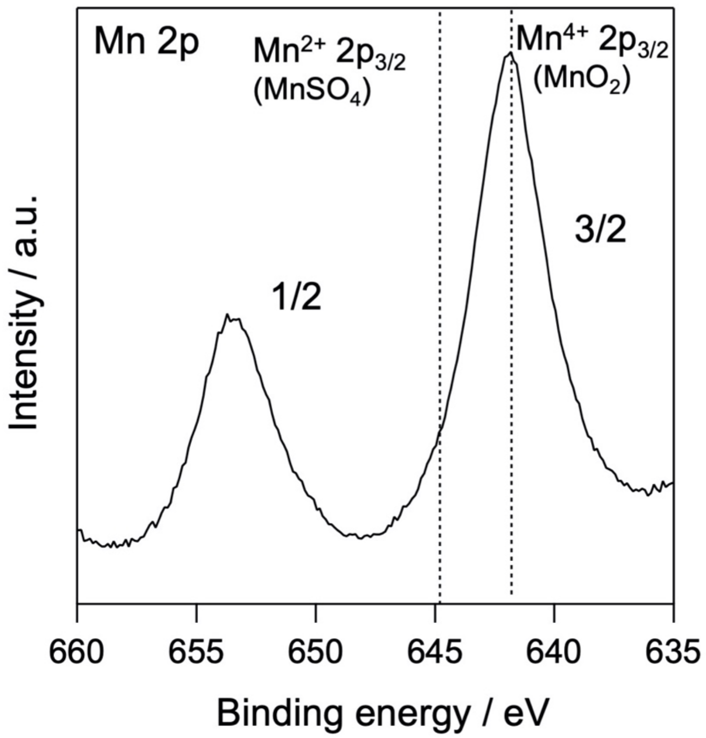

Fig. S31| Valence state of photo-deposited Mn species. XPS spectra of **Rh-Bi_4NbO_8Cl** after the photo-catalytic deposition of MnO_2 in the Mn 2p region. The binding energies of Mn^{2+} and Mn^{4+} were referred to from a reference hand book.[5]

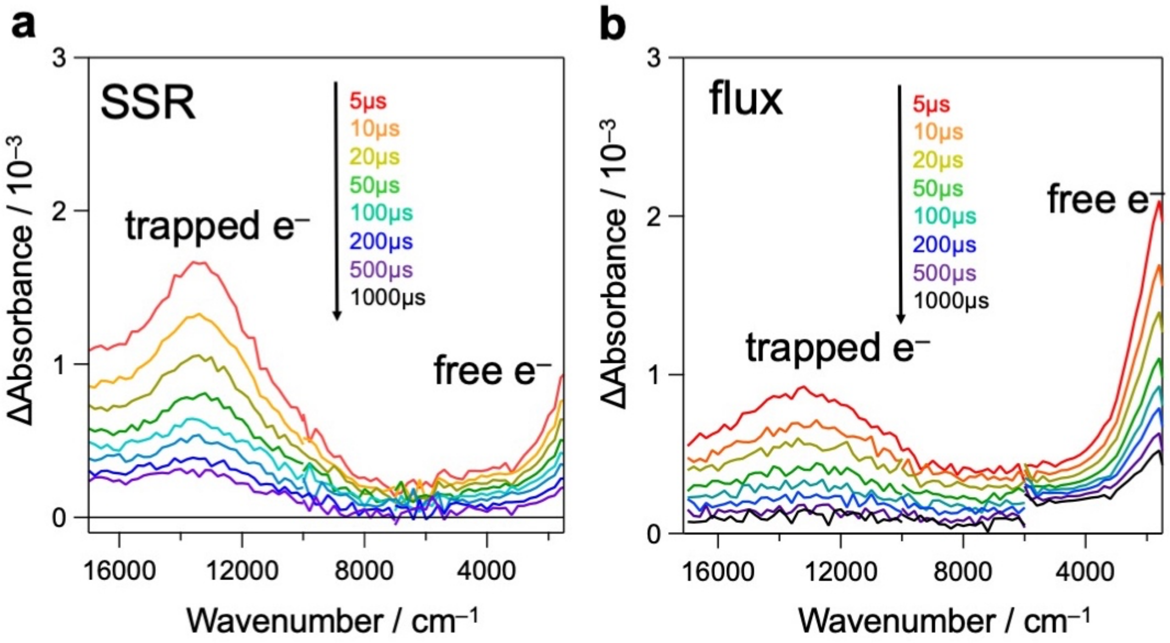

Fig. S32| Time-resolved absorption spectra of Bi_4NbO_8Cl prepared via the conventional solid-state reaction (SSR) and flux synthesis. The samples were excited at 355 nm (6 ns duration, 0.5 mJ, 5 Hz). We previously reported that Bi_4NbO_8Cl prepared by a high temperature solid-state-reaction (SSR) method suffered from Cl defects.[17] In the present study, the Bi_4NbO_8Cl samples prepared via the flux method, where Cl defects are relatively suppressed, were used.[4] In fact, as shown in Figs. 2a, f-l, and Fig. S6, the flux-prepared sample feature high quality of crystals. The signal intensities originating from the trapped electrons (at the mid-gap state) and free ones are, respectively, greater and smaller in SSR-prepared Bi_4NbO_8Cl than flux-prepared one. The TRAS result indicates the mid-gap state should stem from the Cl defects.

References for Supplementary Information

1 H. Kato and A. Kudo, *J. Phys. Chem. B,* 2001, **105**, 4285–4292.

2 S. Zhong, Y. Xi, S. Wu, Q. Liu, L. Zhao and S. Bai, *J. Mater. Chem. A* 2020, **8**, 14863–14894.

3 H. Nishiyama, T. Yamada, M. Nakabayashi, Y. Maehara, M. Yamaguchi, Y. Kuromiya, Y. Nagatsuma, H. Tokudome, S. Akiyama, T. Watanabe, R. Narushima, S. Okunaka, N. Shibata, T. Takata, T. Hisatomi and K. Domen, *Nature*, 2021, **598**, 304–307.

4 K. Ogawa, A. Nakada, H. Suzuki, O. Tomita, M. Higashi, A. Saeki, H. Kageyama and R. Abe, *ACS Appl. Mater. Interfaces*, 2019, **11**, 5642–5650.

5 J. F. Moulder, W. F. Stickle, P. E. Sobol and K. D. Bomben, *Handbook of X-rays photoelectron spectroscopy*, Perkin-Elmer Corporation, Minnesota, 1992.

6 S. Trasatti, *J. Electroanal. Chem. Interfacial Electrochem.*, 1972, **39**, 163–184.

7 R. Konta, T. Ishii, H. Kato and A. Kudo, *J. Phys. Chem. B*, 2004, **108**, 8992–8995.

8 M. Hara, J. Nunoshige, T. Takata, J. N. Kondo and K. Domen, *Chem. Commun.*, 2003, **24**, 3000–3001.

9 G. Hitoki, A. Ishikawa, T. Takata, J. N. Kondo, M. Hara and K. Domen, *Chem. Lett.*, 2002, **31**, 736–737.

10 A. Kasahara, K. Nukumizu, G. Hitoki, T. Takata, J. N. Kondo, M. Hara, H. Kobayashi and K. Domen, *J Phys Chem A*, 2002, **106**, 6750–6753.

11 A. Ishikawa, T. Takata, J. N. Kondo, M. Hara, H. Kobayashi and K. Domen, *J. Am. Chem. Soc.*, 2002, **124**, 13547–13553.

12 K. Ogisu, A. Ishikawa, K. Teramura, K. Toda, M. Hara and K. Domen, *Chem. Lett.*, 2007, **36**, 854–855.

13 A. Romanchenko, M. Likhatski and Y. Mikhlin, *Minerals*, 2018, **8**, 578.

14 Z. Jiang, Z. Zhang, W. Shangguan, M. A. Isaacs, L. J. Durndell, C. M. A. Parlett and A. F. Lee, *Catal. Sci. Technol.*, 2016, **6**, 81–88.

15 C. Tossi, L. Hällström, J. Selin, M. Vaelma, E. See, J. Lahtinen and I. Tittonen, *J. Mater. Chem. A*, 2019, **7**, 14519–14525.

16 T. Tachikawa, T. Yonezawa and T. Majima, *ACS Nano*, 2013, **7**, 263–275.

17 H. Kunioku, A. Nakada, M. Higashi, O. Tomita, H. Kageyama and R. Abe, *Sustain. Energy Fuels*, 2018, **2**, 1474–1480.
